# Reconstructing the Invasion Route of the P-Element in *Drosophila melanogaster* Using Extant Population Samples

**DOI:** 10.1093/gbe/evaa190

**Published:** 2020-09-10

**Authors:** Lukas Weilguny, Christos Vlachos, Divya Selvaraju, Robert Kofler

**Affiliations:** 1 Institut für Populationsgenetik, Vetmeduni Vienna, Wien, Austria; 2 Vienna Graduate School of Population Genetics, Wien, Austria

**Keywords:** P-element, transposable elements, *Drosophila*, population genetics, bioinformatics

## Abstract

The P-element, one of the best understood eukaryotic transposable elements, spread in natural *Drosophila melanogaster* populations in the last century. It invaded American populations first and later spread to the Old World. Inferring this invasion route was made possible by a unique resource available in *D. melanogaster*: Many strains sampled from different locations over the course of the last century. Here, we test the hypothesis that the invasion route of the P-element may be reconstructed from extant population samples using internal deletions (IDs) as markers. These IDs arise at a high rate when DNA transposons, such as the P-element, are active. We suggest that inferring invasion routes is possible as: 1) the fraction of IDs increases in successively invaded populations, which also explains the striking differences in the ID content between American and European populations, and 2) successively invaded populations end up with similar sets of IDs. This approach allowed us to reconstruct the invasion route of the P-element with reasonable accuracy. Our approach also sheds light on the unknown timing of the invasion in African populations: We suggest that African populations were invaded after American but before European populations. Simulations of TE invasions in spatially distributed populations confirm that IDs may allow us to infer invasion routes. Our approach might be applicable to other DNA transposons in different host species.

**Significance**

Transposable elements (TE) are parasites multiplying within genomes. TEs usually rapidly spread within and between populations until, eventually, worldwide populations are infected. Although the invasion routes of TEs are interesting to many research areas little is known about them. Tracing invasions, so far, required the timely discovery of an ongoing TE invasion and recurrent sampling of invaded populations from different geographic locations—severe challenges that were rarely overcome. Here, we introduce a simpler approach. We show that it is feasible to infer the invasion route of the P-element many generations after the spread of the TE using samples from extant populations. As samples from extant populations are becoming increasingly available for many species, our approach will be widely applicable.

## Introduction

Transposable elements (TEs) are short stretches of DNA that selfishly proliferate within host genomes. Most TE insertions likely have negative effects on host-fitness (Mackay 1989; Houle and Nuzhdin 2004; Mackay et al. 1991; Blumenstiel et al. 2014; Yukuhiro et al. 1985). To control the spread of these deleterious elements, host organisms evolved elaborate defense mechanisms. In mammals and many invertebrates, the defense system against TEs relies on piRNAs, small RNAs ranging in size from 23 to 29 nt (Brennecke et al. 2007; Gunawardane et al. 2007). piRNAs associate with PIWI clade proteins, which act to silence TEs with complementary sequences at the transcriptional as well as the posttranscriptional level (Sienski et al. 2012; Le Thomas et al. 2013; Brennecke et al. 2007; Gunawardane et al. 2007). piRNAs are largely derived from discrete genomic loci that have been termed piRNA clusters (Brennecke et al. 2007; Malone et al. 2009). It is assumed that a newly invading TE multiplies within populations until the spread is stopped by TE copies that randomly jumped into piRNA clusters, which triggers the production of piRNAs that silence the TE (Bergman et al. 2006; Malone and Hannon 2009; Zanni et al. 2013; Yamanaka et al. 2014; Goriaux et al. 2014; Duc et al. 2019). Initially, a TE invasion will be stopped by multiple segregating piRNA cluster insertions, but fixed insertions may emerge later on (Kofler 2019; Kelleher et al. 2018).

In spite of these elaborate defense mechanisms, TEs are successful invaders that have been found in most prokaryotic and eukaryotic genomes studied so far (Biémont and Vieira 2006; Wicker et al. 2007). For example, the P-element, one of the best understood eukaryotic TEs, invaded *Drosophila melanogaster* and *D. simulans* within the last 100 years (Kidwell 1983; Kofler et al. 2015; Hill et al. 2016). *D. melanogaster* likely acquired the P-element by horizontal transfer (HT) from the distantly related *D. willistoni* in South America (Daniels and Chovnick 1993). It subsequently spread in worldwide *D. melanogaster* populations between 1950 and 1980 (Anxolabéhère et al. 1985, 1988). The P-element first spread in American populations and later invaded European and African populations (Anxolabéhère et al. 1985, 1988). Reconstructing the invasion route of the P-element was only possible because of the availability of many fly strains sampled during its invasion from different geographic locations (Anxolabéhère et al. 1985, 1988). Here, we test the hypothesis whether it may be feasible to reconstruct the invasion route of the P-element based on extant population samples using internal deletions (IDs) of the P-element as markers. IDs arise at a high frequency during propagation of DNA transposons, such as the P-element. DNA TEs multiply by a “cut-and-paste” mechanism which does not inherently lead to an increase in copy numbers. Instead, the copy number increase is achieved by repair of double-stranded breaks—resulting from excision of the TE—using the sister chromatid as template (Engels et al. 1990; Daniels and Chovnick 1993; Gloor et al. 1991). Interruption of this gap-repair mechanism leads to ID insertions (Engels et al. 1990; Daniels and Chovnick 1993; Gloor et al. 1991). These ID elements are usually nonautonomous and require the enzymes encoded by autonomous full-length (FL) insertions for mobilization (Hua-Van et al. 2011; Wicker et al. 2007). Several properties of IDs can be leveraged for reconstructing invasion routes: First, IDs of many DNA transposons emerge at a high rate. For example, during an experimental P-element invasion, at least 140 different IDs emerged within 60 generations (Kofler et al. 2018). Second, the breakpoints of IDs are mostly random (Kofler et al. 2018). It is thus unlikely that identical pairs of breakpoints will emerge independently multiple times. Third, IDs solely arise when a TE is active (Engels 1989), since IDs of DNA transposons result from interruption of gap-repair after transposition of the TE (Engels et al. 1990; Daniels and Chovnick 1993; Gloor et al. 1991).

We first show that each population invaded by the P-element receives a unique set of IDs, that is, an ID fingerprint. Based on the two insights that the fraction of IDs increases in successively invaded populations and that successively invaded populations share similar ID fingerprints, we inferred an invasion route of the P-element that supports previous studies (Anxolabéhère et al. 1985, 1988). Besides that, our work also provides novel insights into the P-element invasion. Our hypothesis of an increasing fraction of IDs in successively invaded populations provides a simple explanation for the higher fraction of IDs in European than in American populations (as American populations were invaded before European ones) (Black et al. 1987; Bergman et al. 2017). We further suggest that African populations were invaded after American, but before European populations. Finally, we support our approach with simulations of TE invasions under a stepping-stone model. We also note that it may be feasible to extent our approach to other recent invasions of DNA transposons in different host species.

## Results

### Taking ID Fingerprints

**Fig. 1.**
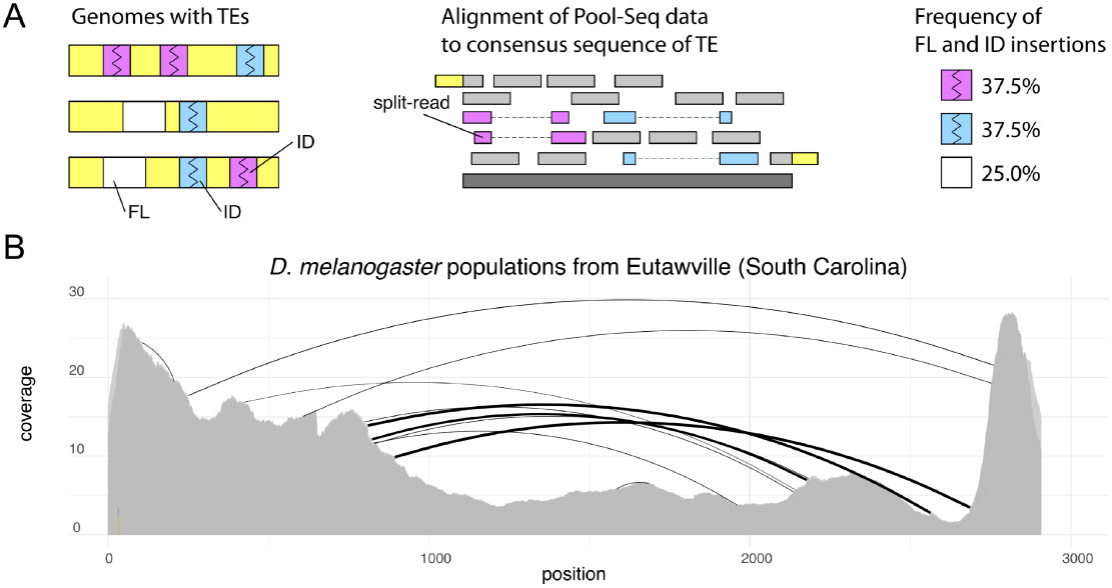
Overview of our approach for taking the ID (internal deletion) fingerprint of a TE. (*A*) A population, represented by a collection of haploid genomes (yellow), harboring some FL (full-length, white) and some ID elements with different breakpoints (blue, magenta; left panel) is sequenced as pool. The reads are aligned to the consensus sequence of a TE family (middle panel), with IDs mapping as split-reads. The frequency of ID and FL insertions can be estimated from the coverage of the TE and the abundance of the split-reads supporting certain IDs (right panel), for example, using our novel tool DeviaTE (Weilguny and Kofler 2019). (*B*) Example of an ID fingerprint for the P-element in a *Drosophila melanogaster* population collected in 2018 from Eutawville (data from Bergland et al. 2014). Arcs indicate the breakpoints of IDs and their width scales with the frequency of the IDs. Three highly abundant IDs (bold arcs) and several less-abundant IDs were found.

Here, we test the hypothesis that the invasion route of the P-element can be reconstructed based on IDs found in extant population samples. This approach requires estimates of the abundance of IDs in the P-element from population samples. For this purpose, we previously developed a novel tool named DeviaTE (Weilguny and Kofler 2019). Briefly, DeviaTE aligns reads of a sample to consensus sequences of TEs (e.g., the P-element) using a local alignment algorithm, reconstructs the breakpoints of IDs from the partial alignment of reads, and finally infers the frequency of IDs by relating the number of reads supporting an ID to the total coverage of the TE (fig. 1*A*; Weilguny and Kofler 2019). As this approach is indifferent to the genomic insertion sites of TEs, a reference assembly is not required. We refer to the set of breakpoints of IDs in a sample, and their respective frequencies as the “ID fingerprint.” As an example, the ID fingerprint of the P-element in a natural population from North America is shown in figure 1*B*.

### Each Invaded Population Receives a Unique ID Fingerprint

**Fig. 2.**
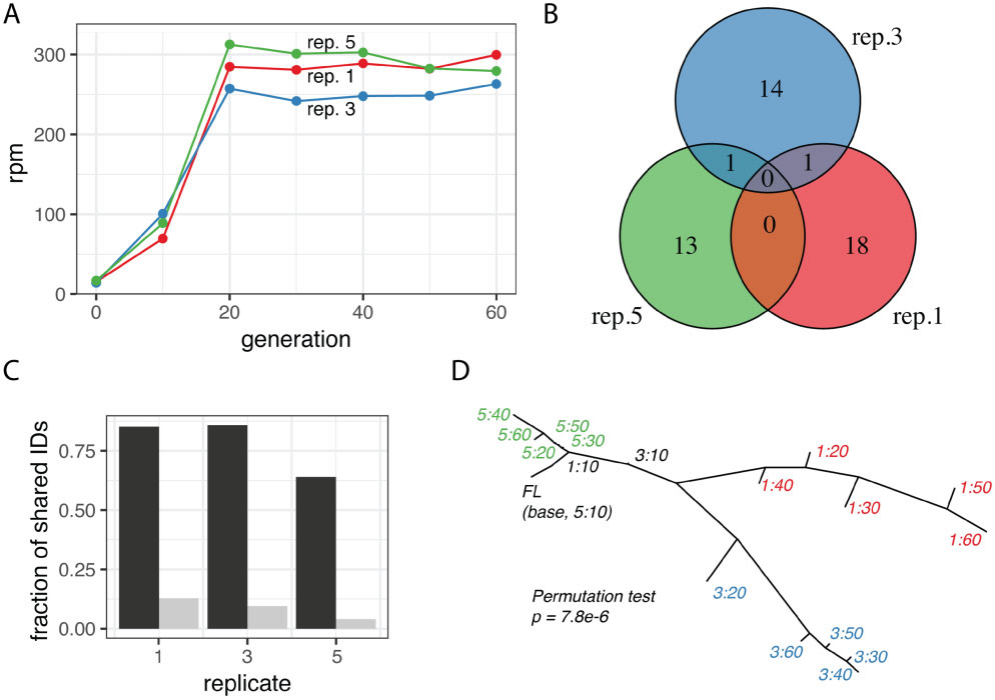
Each population invaded by the P-element receives a unique ID fingerprint. Data are from a replicated P-element invasion in experimental *Drosophila simulans* populations (Kofler et al. 2018). The populations were sequenced each 10th generation as pools. (*A*) In all three replicate populations, P-element copy numbers rapidly increased within the first 20 generations, whereas no further increase was noted for the following 40 generations. (*B*) A Venn diagram of IDs found in different replicates (at any time point) shows that most IDs are replicate specific. (*C*) IDs are more often recovered at different time points of the same replicate (black) than at any time point of a different replicate (gray). Hence, IDs persist within replicates. (*D*) Unrooted tree constructed from the ID fingerprints of the populations. Once the invasions plateaued (≥20 generations), the populations (denoted as “replicate:generation”) lie on replicate-specific clades. A population without IDs (FL, full-length) was included.

Ideally, markers that enable the reconstruction of a TE invasion route should be characteristic to an invaded population and, in the absence of migration, persist within each population. The marker should thus have a high mutation rate during an invasion and a low mutation rate thereafter. To test if IDs of the P-element are suitable markers, we utilized publicly available data of a P-element invasion in experimentally evolving *D. simulans* populations (Kofler et al. 2018). The authors monitored the spread of the P-element in three replicated populations by sequencing the populations at each 10th generation as pools (Kofler et al. 2018). P-element copy numbers rapidly increased for the first 20 generations, whereas no further increase was observed during the next 40 generations (fig. 2*A*). We first tested whether IDs that emerged during the invasions are specific for each replicate population. We solely considered IDs that were supported by at least two split-reads (all IDs were reported in Kofler et al. [2018]) and allowed for a tolerance of 3 bp in the estimated position of ID breakpoints, as exact alignments with indels are frequently not feasible (the position of the gap may be ambiguous). The vast majority of IDs were indeed unique to each replicate (fig. 2*B*; for ID fingerprints, see supplementary fig. 1, Supplementary Material online). Only two out of 47 IDs were found in multiple replicates (fig. 2*B*). These two IDs did not necessarily emerge independently in several replicates but rather might have already been present in the base population (Kofler et al. 2018). Next, we tested if IDs persist within populations. Randomly picked IDs were considerably more often recovered at different time points within the same replicate than at any time point between different replicates (fig. 2*C*). Finally, we reasoned that by treating each ID as an allele of the TE, it may be feasible to calculate a genetic distance among populations that reflects their invasion history. We used Jost’s *D* (Jost 2008) to estimate the genetic distance among samples, and the bionj algorithm to construct a tree from the resulting distance matrix (Gascuel 1997). Jost’s *D* provides unbiased genetic distances when a locus has many alleles (e.g. different IDs) (Jost 2008). Except for the early stage of the invasions, when only very few IDs were found (generation ≤10), all samples were assigned to replicate-specific clades (fig. 2*D*). A permutation test, randomly distributing the 15 samples (≥20 generations) among three different clades (5 samples for each clade) showed that this replicate-specific clustering is highly significant (10^8^ permutations; *P *<* *0.001). SNPs within the P-element provide a less-clear clustering of the samples than the IDs (supplementary fig. 2, Supplementary Material online).

In summary, we showed that each independent P-element invasion generates a unique ID fingerprint that persists within populations. Furthermore, the similarity of ID fingerprints can be quantified, which allows us to calculate genetic distances that reflect the invasion history of samples. Samples from the same invasion are separated by a small distance, whereas samples from independently invaded populations show larger distances.

### IDs Carry Spatial Information

**Fig. 3.**
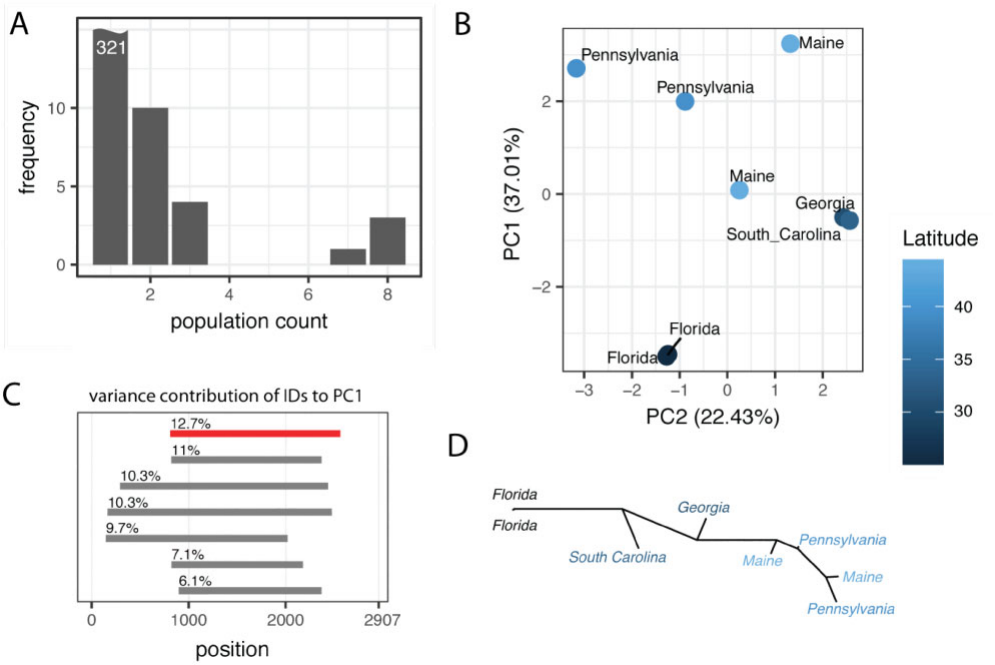
IDs of the P-element recover the latitudinal cline found in *Drosophila melanogaster* populations from the East Coast of the United States (Bergland et al. 2014). (*A*) Distribution of IDs in the populations. Most IDs (321) are only found in a single population, whereas three are found in all eight populations. (*B*) PCA based on the frequency of IDs, with PC1 being correlated with latitude (ρ=0.86, *P *=* *0.0061). (*C*) Variance contribution of the most important IDs to PC1. The start and end positions of IDs (line) as well as the variance contributions of IDs are shown. The KP-element is shown in red. (*D*) Unrooted tree based on the genetic distance of the ID fingerprints. A latitudinal cline can be observed along the tree.

Next, we asked whether IDs are useful markers for spatial population genetics. Using publicly available data, we asked whether IDs of the P-element allow us to reproduce spatial signals found in previous works that relied on SNPs as markers. Some similarities in the spatial signal of SNPs and IDs may be expected, assuming that the migration pattern which shaped genome-wide polymorphism of SNPs also influenced the distribution of IDs. Bergland et al. (2014) found a latitudinal cline in sequenced *D. melanogaster* populations from the East Coast of North America (“Bergland data”) and Kapun et al. (2020) report a longitudinal cline in populations from different locations in Europe (“DrosEU data”). In both data sets, several IDs were found in more than one population (18 for Bergland; 124 for DrosEU; fig. 3*A*;  supplementary fig. 3*A*, Supplementary Material online). However, a large fraction of the IDs are solely found in a single population (321 and 572 for the Bergland and DrosEU data, respectively). Such a large fraction of population-specific IDs may be a source of noise when assessing the genetic distance among populations based on IDs. In agreement with this, the spatial autocorrelation was significant for both data sets when population-specific IDs were excluded from the analysis, but not when they were included (Mantel permutation test, 100,000 permutations; geographic distance vs. genetic distance based on ID fingerprints; Bergland data $P_{\text{incl}.}=0.857,\;P_{\text{excl}.}=0.0103$; DrosEU data $P_{\text{incl}.}=0.0807$, *P*_excl_=0.00001). Simulations confirmed that exclusion of these population-specific IDs allows us to estimate the genetic distance among populations more accurately (supplementary text 1, Supplementary Material online). In all following analyses, we thus ignored population-specific IDs for estimating the relationship among populations with principal component analysis (PCA) and Jost’s *D*.

We performed a scaled PCA with the frequency of IDs found in the Bergland data (fig. 3*B*). Note that all IDs have identical variance contributions after scaling. PC1 was significantly correlated with latitude (Spearman’s rank correlation ρ=0.86, *P *=* *0.0061) and weakly correlated with longitude (Spearman’s rank correlation ρ=0.74, *P *=* *0.036). No correlation with either longitude or latitude was found for PC2 (Spearman’s rank correlation; latitude ρ=−0.21, *P *=* *0.62; longitude ρ=−0.01, *P *=* *0.98). As the KP-element, an ID where nucleotides 808–2,560 of the P-element are deleted, is widespread and highly abundant in worldwide populations of *D. melanogaster* (Black et al. 1987; Itoh and Boussy 2002; Bergman et al. 2017), we asked whether the PCA results were mainly due to the KP-element. Investigating the contribution of the different IDs to PC1, we found that the KP-element indeed had the strongest influence on PC1, but several other IDs had very similar effects (fig. 3*C*;KP-element shown in red). We also repeated the PCA without the KP-element and found that the significant correlation of PC1 with latitude could still be observed (Spearman’s rank correlation; ρ=0.80, *P *=* *0.017). The observed latitudinal cline is therefore due to frequency variation of multiple IDs. Finally, we used the genetic distance among ID fingerprints to generate an unrooted tree (fig. 3*D*). The resulting tree also reflects the latitudinal cline, with samples being largely sequentially ordered from the South (Florida) at one end to the North (Maine) at the other end of the tree (fig. 3*D*).

Next, we conducted a scaled PCA with the frequency of IDs found in the DrosEU data (supplementary fig. 3*B*, Supplementary Material online). Both PC1 and PC2 showed only minor contributions to the total variance (PC1=9.51%, PC2=6.8%; supplementary fig. 3*B*, Supplementary Material online). However, both PC1 and PC2 were significantly correlated with longitude but not with latitude (Spearman’s rank correlation; longitude $\rho_{\text{PC}1}=-0.81,\;P_{\text{PC}1}<0.001,\;\rho_{\text{PC}2}=-0.55,\;P_{\text{PC}2}<0.001$; latitude $\rho_{\text{PC}1}=0.21,\;P_{\text{PC}1}=0.15,\;\rho_{\text{PC}2}=0.003,\;P_{\text{PC}2}=0.98$;). Again, the KP-element had a minor contribution to PC1 (supplementary fig. 3*C*, Supplementary Material online). A scaled PCA excluding the KP-element confirms the correlation of PC1 and PC2 with longitude but not with latitude (Spearman’s rank correlation; longitude $\rho_{\text{PC}1}=-0.82,\;P_{\text{PC}1}<0.001,\;\rho_{\text{PC}2}=-0.57,\;P_{\text{PC}2}<0.001$; latitude $\rho_{\text{PC}1}=0.22,\;P_{\text{PC}1}=0.12,\;\rho_{\text{PC}2}=0.05,\;P_{\text{PC}2}=0.72$).

We conclude that IDs of the P-element allow us to reproduce spatial information described in previous works (longitudinal cline in DrosEU data and latitudinal cline in Bergland data). IDs of the P-element may therefore be useful markers for spatial population genetics.

### Dynamics of TE Invasions with IDs

We performed simulations to test whether IDs may be used to infer the invasion route of TEs. These simulations have two main goals. Firstly, we want to show that the fraction of IDs allows us to estimate the direction of an invasion. And secondly, we aim to show that successively invaded populations may end up with similar ID fingerprints, which permits the inference of the path of an invasion. The path of the invasion combined with the direction of the invasion allows us to infer the invasion route. Note that we do not aim to accurately reproduce the invasion dynamics of the P-element in spatially distributed populations due to our limited knowledge about the TE dynamics and the uncertainty about many key parameters. These parameters include the migration patterns among populations, the sizes of the various subpopulations, the extent of selection on P-element insertions and IDs, the rate at which IDs emerge, the transposition rate, the speed of silencing of the P-element, and the extent of various biases, such as preferential mobilization of ID elements, the insertion bias into piRNA clusters or biases in the position of ID breakpoints.

**Fig. 4.**
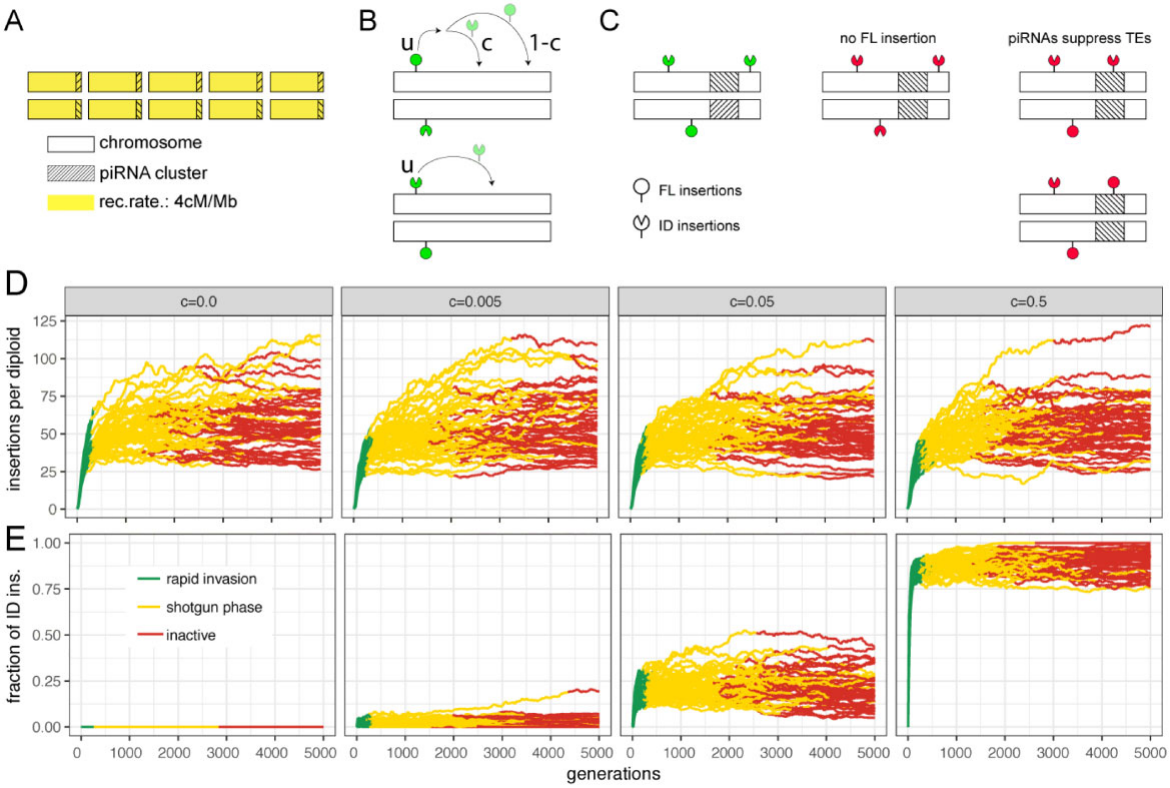
Dynamics of TE invasions with IDs. (*A*) We simulated diploid organisms with five chromosomes of 1-Mb harboring piRNA clusters of 100 kb at one end of each chromosome. (*B*) Although both FL and ID insertions are mobilized with a transposition rate *u*, only FL elements may be converted to ID elements (with probability *c* per transposition event). (*C*) In our model, TEs are active (green) in individuals that have at least one FL insertion but no insertion (FL or ID) in a piRNA cluster. An absence of FL insertions or a piRNA cluster insertion leads to inactive TEs (red). (*D*) Influence of the conversion rate (*c*; top panel) on the TE abundance during an invasion. Data are shown for 50 replicates. The conversion rate has a negligible effect on the TE abundance. (*E*) Fraction of ID insertions during a TE invasion. Note that the abundance of ID insertions solely increases at early stages of an invasion (green).

Initially, we investigated the dynamics of TE invasions with IDs in a single population and later extend this model to multiple populations with migration. We simulated diploid organisms with five chromosomes of 1 Mb, a recombination rate of 4 cM/Mb, and piRNA clusters of 100 kb at one end of each chromosome (fig. 4*A*). We modeled the spread of a neutral TE with a given transposition rate (*u*) in a population of size *N *=* *1,000 (fig. 4*B*). The TE invasions were launched by randomly distributing 300 FL elements in the genome (population frequency of insertions 1/2N). We assumed that IDs result from an interruption of sister chromatid-mediated gap repair following excision of an FL element (Engels et al. 1990; Daniels and Chovnick 1993; Gloor et al. 1991). Hence, IDs are solely generated when the TE is active. A transposing FL element yields a novel ID with probability *c* (henceforth “conversion rate”; fig. 4*B*; top panel). For each novel ID, we randomly picked two breakpoints. We assumed that FL and ID elements transpose at the same rate *u* and that each copy may only harbor a single ID, that is, a TE insertion with an ID cannot accumulate further IDs (fig. 4*B*). ID elements are assumed to be nonautonomous (Hua-Van et al. 2011), therefore mobilization of ID elements requires the presence of at least one FL insertion per diploid individual (fig. 4*C*). Finally, we simulated TE invasions under the trap model, which holds that the proliferation of an invading TE is stopped by TEs (FL or ID) that randomly jump into piRNA clusters (Kofler 2019; Kelleher et al. 2018). The TE is thus only active in diploid individuals that do not carry a cluster insertion (fig. 4*C*, left). We assumed that both ID and FL insertions in piRNA clusters inactivate a TE, as previous works demonstrated that short stretches of homology between a cluster insertion and the TE sequence are sufficient for piRNA-mediated silencing (Post et al. 2014).

The dynamics of TE invasions with piRNA clusters have been explored before (Kelleher et al. 2018; Lu and Clark 2010; Kofler 2019). In the absence of IDs (*c *=* *0.0), we found the typical three phases of a TE invasion (fig. 4*D*; Kofler 2019). First, the TE rapidly spreads in invaded populations (rapid invasion phase, green), then the TE is silenced by segregating cluster insertions (shotgun phase, yellow), and finally, the TE is permanently inactivated by a fixed cluster insertion (inactive phase, red; fig. 4;  Kofler 2019). Introducing IDs into our model (*c *>* *0) had little influence on the invasion dynamics (fig. 4*D*). The length of the phases as well as the TE abundance were largely unaffected by IDs (supplementary fig. 4, Supplementary Material online). However, the conversion rate (*c*) had a profound effect on the abundance of ID elements (fig. 4*E*). The fraction of ID insertions increased rapidly during the early stages of an invasion (green) but plateaued as soon as the TE was silenced (yellow, red; fig 4*E*). The abundance of IDs at the plateau depended on the conversion rate (*c*), as, for example, 7% of TEs ended up with an ID when *c *=* *0.005 and 25% when *c *=* *0.05 (fig. 4*E*). Importantly, the fraction of IDs did not increase any further after the TE was silenced (fig. 4*E* and  supplementary fig. 5, Supplementary Material online). This raises the question how worldwide populations may end up with vastly different numbers of P-element IDs, with populations from North America harboring few IDs, whereas populations from Europe have many IDs (Bergman et al. 2017; Black et al. 1987).

**Fig. 5.**
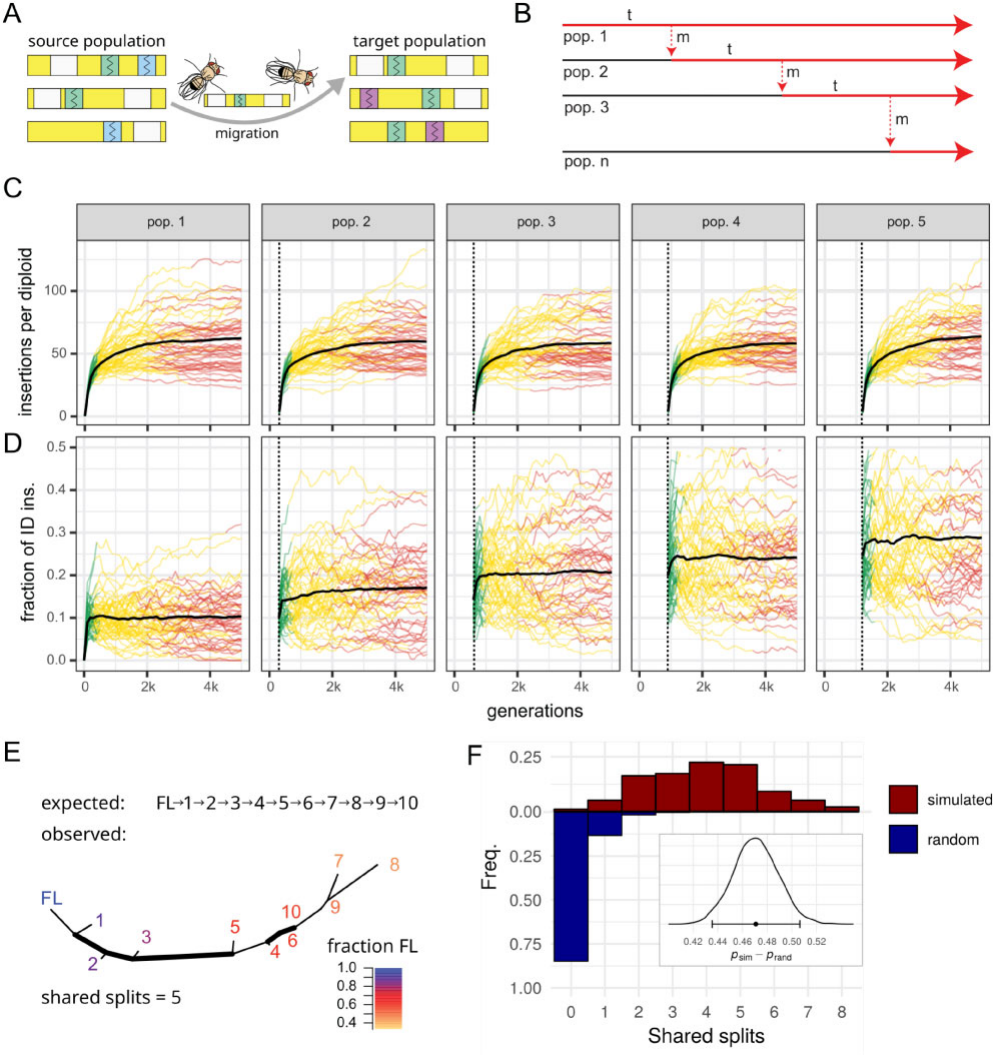
Model for reconstructing the spatial invasion history of TEs. (*A*) Migrants from a source population (genomes shown in yellow) carry some IDs (colored boxes) and FL insertions (white boxes) to a target population, thereby triggering a novel invasion. During the invasion of the target population, IDs introduced by migrants will be propagated (green) and novel IDs will be acquired (violet). (*B*) Stepping-stone model of successive TE invasions. After a TE invaded a population (*t *=* *300), a number of migrants (red dashed line, *m *=* *100) move to a naive population, not having the TE (black line), thereby triggering a new invasion (red line). (*C*) TE abundance in successively invaded populations (top panel). The dashed lines indicate migration events introducing the TE and bold lines indicate the average across 50 simulations. (*D*) Fraction of ID insertions in successively invaded populations. Note that the fraction of IDs increases with each invaded population. (*E*) Example of a reconstructed TE invasion. Based on ID fingerprints, a distance matrix and a tree were constructed. Numbers refer to the order of the invaded populations. As origin of the invasion a FL insertion was included (FL). Note that the fraction of FL insertions decreases (hence the abundance of IDs increases) with each invaded population. The tree shares five splits (bold) with the expected one. (*F*) Accuracy of reconstructed TE invasions for 100,000 random trees and 100 trees derived from our simulations. In this scenario, the highest possible number of shared splits is eight. The difference in posterior probabilities for observing a shared split (inset) shows that zero is not in the ${\text{CI}}_{95\%}$ and our approach thus captures substantially more information than random trees. Error bars indicate the ${\text{CI}}_{95\%}$.

Here, we propose a simple explanation for the varying abundance of P-element IDs in worldwide populations, which also highlights an approach for estimating the direction of an invasion as a side effect. We suggest that individuals migrating from an invaded population to a non-invaded population will introduce a sample of the insertions (FL and IDs) from the source into the target population. This will trigger a novel invasion in the target population (fig. 5*A*). Both FL and IDs introduced by the migrating individuals will increase in copy numbers during the invasion. Additionally, however, novel IDs will be acquired due to the renewed TE activity (fig. 5*A*). The target population will therefore, on average, end up with more IDs than the source population (fig. 5*A*). The fraction of IDs will increase in successively invaded populations and thus serve as a guide to the direction of an invasion. Accordingly, the origin of an invasion should be close to the population with the fewest IDs. We tested this hypothesis using a stepping-stone model with five populations and a conversion rate of *c *=* *0.025 (fig. 5*B*). We initiated an invasion in the first population using 250 randomly distributed FL elements and allowed TEs to invade a population for 300 generations before introducing the next migration event. This enabled populations to acquire distinct, stable ID fingerprints (most TEs were silenced by generation 300). After 300 generations, 100 migrants were moved from the invaded population to the next naive population, thus triggering a novel invasion (fig. 5*B*). We repeated these migration events until all populations were infected by the TE (after 1,200 generations). Under our model, the abundance of TEs remained constant in successively invaded populations (fig. 5*C* and  supplementary fig. 6, Supplementary Material online). However, the fraction of IDs significantly increased with each successively invaded population (fig. 5*D* and  supplementary fig. 6, Supplementary Material online). This confirms that the fraction of IDs serves as a rough guide to the direction of an invasion, where populations that were invaded first contain the fewest IDs. Furthermore, this may also explain why P-elements in North American populations have fewer IDs than in European populations (Bergman et al. 2017; Black et al. 1987). As North American populations were invaded first, they will harbor fewer IDs than the subsequently invaded European populations (Anxolabéhère et al. 1988).

Next, we tested whether we can infer the path of an invasion based on the similarity of ID fingerprints among populations. Using the stepping-stone model introduced above, we simulated TE invasions in ten successive populations and used Jost’s *D* to estimate the similarity of ID fingerprints among populations after 3,000 generations, that is, after all ten populations were invaded (the last migration event occurred at generation 2,700). This results in a matrix of pairwise distances among populations. Additionally, to mark the origin of the invasion, we included an artificial population consisting solely of FL elements into this distance matrix (for an overview of simulation parameters see supplementary table 1, Supplementary Material online). Finally, we inferred trees from the distance matrices and compared them to the expected tree (with the expected invasion route: FL → 1 →… → 10). We assessed the accuracy of the inferred trees using the number of splits shared between the observed and the expected tree. A split, or bifurcation, is the smallest information unit of unrooted trees and the number of splits shared between two trees may be used as a similarity metric when only the topology of trees is considered (i.e., the branch length is ignored). In our scenario, the largest possible number of shared splits is 8. As an example, a tree with five shared splits already enables fairly accurate inference of the invasion route (fig. 5*E*). To construct a null expectation, we computed the shared splits between 100,000 random trees and the expected tree. Trees obtained from our simulations had a substantially higher information content than random trees ($P_{\text{rand}}=0.021,\;{\text{CI}}_{95\%}[0.020,0.022];P_{\text{sim}}=0.492,\;{\text{CI}}_{95\%}[0.46,0.53]$, fig. 5*F*). Finally, we evaluated the influence of multiple parameters on the accuracy of the inferred invasion routes: The sampling time and its heterogeneity, the size of piRNA clusters (which determines the TE abundance), the migration rate, the migration pattern, the time between migration events, the conversion rate, the test statistic for computing genetic distances, and preferred mobilization of IDs (supplementary text 1, Supplementary Material online). Under our model, the most important parameters were the conversion rate, the size of piRNA clusters (= abundance of TEs), and the migration pattern (supplementary text 1, Supplementary Material online).

In summary, we showed that it may be feasible to infer the invasion route of DNA transposons using IDs as markers. The similarity of ID fingerprint provides cues about the invasion path and the abundance of FL insertions acts as a rough guide to the direction of an invasion.

### Reconstructing the P-Element Invasion

>Next, we applied our approach to worldwide populations of *D. melanogaster* to test if the reconstructed invasion route of the P-element agrees with previous work. The global P-element invasion in *D. melanogaster* likely started in South America following a horizontal transfer from *D. willistoni* (Daniels and Chovnick 1993). The P-element first spread within South and North American populations, and later in European and African populations (Anxolabéhère et al. 1988). It is, however, not clear whether European or African populations were invaded first. In Africa the P-element was first observed in the South, whereas in Europe the P-element was first found in France (Anxolabéhère et al. 1985, 1988). Starting from France the P-element spread to Spain and towards the East of Europe (Anxolabéhère et al. 1985, 1988).

**Fig. 6.**
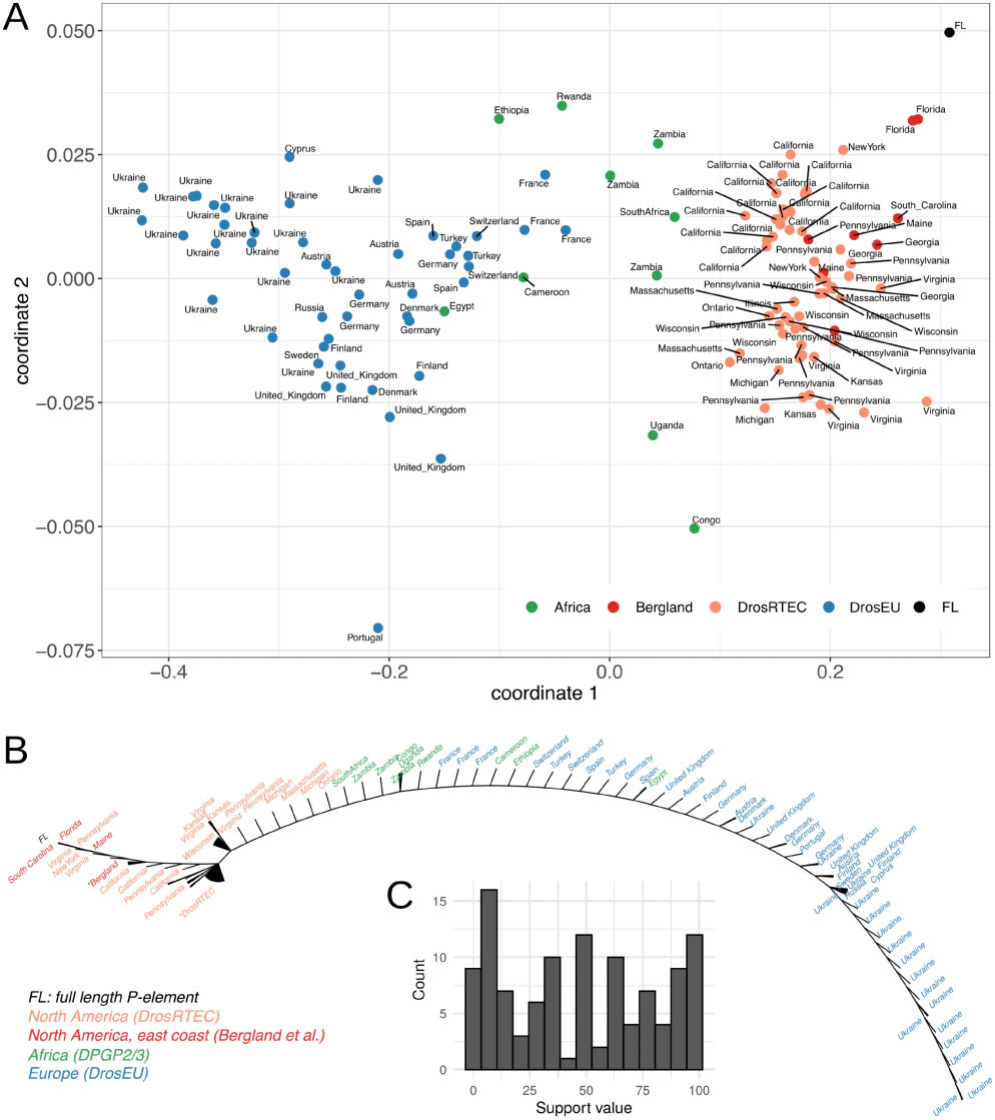
Reconstructed invasion route of the P-element in worldwide populations of *Drosophila melanogaster*. (*A*) Multidimensional scaling (MDS) plot based on the similarity of ID fingerprints among populations. Note that African samples cluster between North American and European samples. (*B*) Consensus tree showing the invasion route of the P-element. Starting from the FL insertion, the P-element first invaded North America, then spread from southern to northern Africa and finally invaded Europe from West to East. This consensus tree is based on 100 bootstrapped trees generated by randomly sampling IDs with replacement. Nodes with <50% support value are collapsed (triangular branches). * indicate multiple samples from the same data set. (*C*) Distribution of bootstrap support values for the nodes of the inferred invasion route (*B*).

To infer the invasion route of the P-element, we relied on the Bergland and DrosEU data mentioned above (Kapun et al. 2020; Bergland et al. 2014), as well as the DrosRTEC data from North America (Machado et al. 2018) and the DPGP2/3 data from Africa (Lack et al. 2015). To avoid biases, we discarded reads smaller than 90 bp and trimmed all reads to a size of 100 bp. For an overview of the abundance of FL and ID P-elements in these populations, see supplementary figure 7, Supplementary Material online. The prevalence of IDs in these samples can be found in supplementary figure 8, Supplementary Material online. We estimated the similarity of ID fingerprints among populations using Jost’s *D* and visualized the resulting distance matrix with a multidimensional scaling (MDS) plot and a consensus tree, based on 100 trees generated by bootstrapping IDs (fig. 6). Neighboring samples in the MDS plot have similar ID fingerprints. An artificial population containing solely FL elements was added to mark the likely origin of the invasion (fig. 6).

Based on our approach, we suggest that the P-element invaded populations from Florida and California first and then spread into other North American populations (fig. 6). Interestingly, our data suggest that the P-element invaded African populations prior to European ones (fig. 6 and supplementary fig. 9, Supplementary Material online). Within Africa, the P-element spread from South to North, until it eventually invaded Europe (fig. 6). Starting from France, the P-element spread to Spain and towards the East of Europe. Lastly, populations from Ukraine were invaded (fig. 6).

The invasion route of the P-element inferred by our approach is remarkably similar to the route proposed by previous works, which relied on fly strains sampled over the course of decades from different geographic locations (Anxolabéhère et al. 1985, 1988). It may thus be feasible to reconstruct the invasion route of the P-element (and possibly other DNA TEs) using IDs as markers

## Discussion

### Using IDs as Genetic Markers

In this work, we propose that IDs allow us to trace the invasion route of the P-element. This is made possible by an interesting property of IDs of DNA transposons: They emerge at a high rate solely when the TE is active (Engels et al. 1990; Daniels and Chovnick 1993; Gloor et al. 1991). As a consequence, each independently invaded population receives a unique ID fingerprint, which remains recognizable over multiple generations (fig. 2). Furthermore, we found that IDs of DNA transposons are useful markers for spatial population genetics. For example, IDs of the P-element support the previously described clines in *D. melanogaster* populations based on genome-wide SNP data (Machado et al. 2018; Kapun et al. 2020). Interestingly, clines in populations from Europe and North America could not be observed when other properties of TEs, such as TE abundance or TE diversity were investigated (Adrion et al. 2019; Lerat et al. 2019). We thus think that IDs are sensitive markers that make it possible to pick up spatial signals that are otherwise not found with TEs.

Interestingly, many IDs were only found in a single population (fig. 3 and supplementary fig. 3, Supplementary Material online). In Europe, for example, 527 out of 696 IDs (75%) were specific to individual populations (supplementary fig. 3, Supplementary Material online). This is in agreement with our simulations where 74% of IDs are found in a single population. The large fraction of population-specific IDs may thus be due to migrants carrying only a small sample of IDs from the source to the target populations. Many novel population-specific IDs may emerge in the target population during the invasion triggered by the migrants. Alternatively, the large number of population-specific IDs may be due to ongoing P-element activity, as novel population-specific IDs may still emerge when the P-element is active. However, we deem this scenario unlikely. In a previous work, we found that a piRNA-based defense mechanism against an invading P-element rapidly emerged in *D. simulans* (Kofler et al. 2018). Within a mere 20 generations, P-element proliferation was stopped in all investigated replicates (Kofler et al. 2018). The P-element invaded worldwide *D. melanogaster* populations several hundred generations ago (1950–1980 with 15 generations per year; Anxolabéhère et al. 1988; Pool 2015), which should be sufficient time to establish a piRNA-based defense against the P-element.

Homoplasic IDs, that is, IDs emerging independently in different populations, could limit the utility of IDs as markers. However, the large numbers of IDs specific to single replicates of the experimental populations and to individual natural populations suggest that homoplasy is not widespread for IDs of the P-element (figs. 2 and 3; supplementary fig. 3, Supplementary Material online). Positive or negative selection of IDs may also affect the utility of IDs as markers (Black et al. 1987; Srivastav et al. 2019).

### Model Assumptions

Our approach for reconstructing the invasion route of DNA transposons rests on two assumptions. First, that the abundance of IDs in populations provides cues about the direction of an invasion, as the fraction of IDs increases in successively invaded populations. Second, that we may infer the path of an invasion from the similarity of ID fingerprints among populations. Our hypothesis that early invaded populations end up with the most FL elements, may be regarded as somewhat counterintuitive. It could be argued that early invaded populations should have the fewest FL elements, since the TE was active for the longest time in these populations allowing them to accumulate most IDs (Bergman et al. 2017). However, this view does not consider that TE invasions are rapidly silenced by segregating piRNA cluster insertions (Kofler 2019; Kofler et al. 2018; Kelleher et al. 2018) and that no further increase in the fraction of IDs is expected once the TE is silenced (supplementary fig. 5, Supplementary Material online). The fraction of IDs may solely increase if a silenced TE is reactivated by some means. Here, we propose that a TE may be reactivated by migrants that trigger a new invasion in a naive population (fig. 5). During this invasion, IDs introduced by migrants will be amplified and novel IDs will emerge. As a consequence, the fraction of IDs will increase in successively invaded populations (supplementary fig. 6, Supplementary Material online). Our model thus provides an explanation for the striking differences in the abundance of P-element IDs between populations from Europe and North America (Black et al. 1987; Bergman et al. 2017). As European populations have more IDs than North American ones, our model predicts that Europe was invaded after North America, which is in agreement with a previous study (Anxolabéhère et al. 1988). Alternatively, it was proposed that North American populations may be able to control IDs more efficiently than European populations (Bergman et al. 2017). This model, however, has two shortcomings: It requires a hitherto unknown mechanism for controlling IDs; and it does not explain how differences in the regulation of IDs among continents may emerge.

Our simulations show that the invasion path may be inferred from the similarity of ID fingerprints of the populations. According to our model, the conversion rate, the size of piRNA clusters (and thus the TE abundance), and the migration pattern have the strongest influence on the accuracy of the inferred invasion path (supplementary text 1, Supplementary Material online). Reconstructing invasion routes may therefore only work for TEs and species that meet certain requirements (e.g., a minimum conversion rate at which IDs emerge). It is likely that other factors, not evaluated in our simulations, also influence the accuracy of our approach, such as the population size, homoplasy, selection acting on IDs, deletion biases, and the number of sampled individuals. Finally, our model is based on the current understanding of TE dynamics (Lu and Clark 2010; Kelleher et al. 2018; Kofler 2019), which is, however, still rather limited. For example, we assumed that random insertions into piRNA clusters stop TE invasions, which makes silencing of TE invasions a lengthy process. Our simulations suggest that up to 300 generations are necessary to stop a TE invasion (fig. 4; see also, Kofler [2019]). However, in experimental populations, silencing of the P-element required a mere 20 generations (Kofler et al. 2018). The reason for this discrepancy is unknown. One possible explanation is that an insertion bias of the P-element into piRNA clusters (Kofler et al. 2018; Zhang et al. 2020; Karpen and Spradling 1992) accelerates silencing of the TE. It is even feasible that piRNA clusters are not necessary to trigger the production of piRNAs against a TE and thus to stop an invasion. For example, Yu et al. (2019) proposed that the emergence of piRNAs against KoRV in koala (a retrovirus that is currently becoming endogenous) is triggered by unspliced TE transcripts. Due to our lack of knowledge about the dynamics of TE invasions, especially in spatially distributed populations, we caution against overinterpreting the simulation results. Nevertheless, the simulations suggest that: 1) the fraction of IDs is expected to increase in successively invaded populations and 2) that it may be feasible to reconstruct TE invasions using IDs as markers, at least under certain conditions.

### P-Element Invasion

To trace the invasion of the P-element, we applied our approach to available sequencing data from *D. melanogaster* populations. The inferred invasion route agrees remarkably well with previous works (Anxolabéhère et al. 1985, 1988; Periquet et al. 1989; Bonnivard et al. 2000). Based on the frequency of IDs, we suggest that African populations were invaded between North American and European populations. We, however, note that Bergman et al. (2017) found similar frequencies of IDs between populations from Europe (Montpellier, France) and Africa (Accra, Ghana) which does not support this scenario. In this work, we solely investigated geographic regions for which multiple samples were available to reduce the influence of possible outlier populations (i.e. we used populations from North America, Europe, and Africa). It would be interesting to extend our approach to populations from Central Asia and South America. As South America is the likely origin of the P-element invasion (Daniels et al. 1990), populations from South America ought to have—according to our model—most FL elements. Central Asian populations are of interest since they may potentially lack the P-element. We found that the fraction of FL elements decreased in Europe from West to East, where very few FL elements were detected in Ukrainian populations (supplementary fig. 7, Supplementary Material online). As P-element activity requires autonomous FL insertions, the continuous dilution of FL elements from West to East raises the intriguing possibility that the P-element invasion may have died down in the East of Europe due to a shortage of autonomous FL insertions. Some TE invasions may thus have a maximum range, beyond which the TE cannot spread due to the lack of FL elements.

### Outlook

We showed that an approach based on sequencing extant populations as pools, taking ID fingerprints of TEs with DeviaTE and computing their similarity with Jost’s *D* allows reconstructing the P-element invasion with reasonable accuracy. In principle, this approach may be extended to other DNA transposons. Reconstructing the invasion route of hobo, a DNA transposon that invaded *D. melanogaster* populations before the P-element, however, led to less-clear results, possibly because the hobo invasion happened earlier than the P-element invasion (supplementary text 2, Supplementary Material online). Our method requires sequences of DNA TEs of interest and sequencing data of populations (or strains) from different geographic locations but no genome assemblies. Therefore, it could be used with model as well as non-model organisms. Due to the efforts of international consortia and individual research groups, population data from different geographic regions will increasingly become available for many diverse species (Kapun et al. 2020; Machado et al. 2018; Alonso-Blanco et al. 2016; Telenti et al. 2016). Hence, it may soon be feasible to extend our approach to different species.

## Materials and Methods

### Publicly Available Data

To analyze IDs during a P-element invasion in *D. simulans*, we used the data of Kofler et al. (2018). Further, we analyzed Pool-Seq data of natural *D. melanogaster* populations sampled from Europe (DrosEU; Kapun et al. 2020), North America (DrosRTEC; Machado et al. 2018), the East Coast of North America (Bergland et al. 2014), and Africa (DPGP2/3; Lack et al. 2015). From the data of Bergland et al. (2014), we used the samples with the most consistent sampling time when multiple samples were available from one location (Linvilla 2009: SRR1525768, SRR1525769). From DPGP2/3 data, we only used samples of complete genomes with a minimum read length of 100 bp and >1,000,000 reads. Solely sequences of individual strains were available in the DPGP2/3 data. Therefore, we artificially pooled them by sampling equal numbers of reads from strains with identical geographic origins. For an overview of all samples used in this work, see supplementary table 2, Supplementary Material online.

### Analysis of Data

All genomic data were downloaded using SRA-Tools (http://ncbi.github.io/sra-tools/, last accessed October 8, 2020) and aligned to a reference consisting of the consensus sequences of TEs from *D. melanogaster* (v9.44; https://github.com/bergmanlab/transposons, last accessed October 8, 2020; Quesneville et al. 2005) and sequences of the single-copy genes *Rhino*, *RpL32*, and *traffic jam*. The single-copy genes were used to estimate TE copy numbers with DeviaTE (Weilguny and Kofler 2019). To avoid heterogeneous read lengths, we trimmed all reads to a length of 100 bp. Reads were mapped with bwasw (Li and Durbin 2009) and processed with samtools (Li et al. 2009). Subsequently, the position and frequency of IDs were estimated using DeviaTE (Weilguny and Kofler 2019). A custom script (*dm-deviate.py*) was used to estimate the genetic distance among samples. We treated each ID and the FL insertion as an allele of a TE and used Jost’s *D* (Jost 2008) to compute the genetic distance among samples. The frequency of the FL insertion was computed as $1-\sum f_{i}$, where *f_i_* are the frequencies of IDs. As alignments of reads with indels are difficult, we allowed for a tolerance of 3 bp in the position of breakpoints. We required a minimum support of 2 reads for each ID in the data of Kofler et al. (2018) and 3 reads in data from natural *D. melanogaster* populations (since few reads map to the P-element in the data of Kofler et al. [2018]). Finally, IDs solely occurring in a single population were ignored for PCA and estimates of Jost’s *D* (increasing the accuracy of our approach). Distance matrices based on Jost’s *D* were used to generate MDS plots and unrooted trees with the bionj algorithm (Gascuel 1997) implemented in the R package ape (Paradis et al. 2004). The majority-rule consensus tree of the P-element invasion was constructed from 100 bionj trees after bootstrapping IDs using ape (Paradis et al. 2004). PCA was performed with the frequency of IDs using R (prcomp; R Core Team 2014). The geographic distance among samples was estimated with the R package geosphere (Hijmans 2017) and the Mantel test for estimating the correlation of distance matrices was performed with the R package cultevo (Stadler 2018).

### Simulations

Simulations were performed with a modified version of the Java tool Invade (Kofler 2019). The adapted tool invade-td (*t*runcations *d*eme) performs individual-based forward simulations of TE invasions with piRNA clusters and IDs under a 1D stepping-stone model (Kimura and Weiss 1964). At each generation, the tool performs the following steps in the given order: 1) mate pairs are formed based on the fitness of the individuals, 2) haploid gametes are generated based on the recombination map, 3) novel FL and ID insertions are introduced, 4) zygotes are formed, 5) piRNA cluster insertions are counted, 6) the fitness of the individuals is computed, 7) migrants are exchanged between adjacent populations (optional), and 8) the output is produced (optional). Novel IDs emerged during the transposition phase (3) with probability *c* per transposing FL element. Two random breakpoints were chosen for each novel ID (between positions 1 and 2907). If not mentioned otherwise, we simulated a genome with five chromosomes of 1 Mb (–genome Mb:1,1,1,1,1), a recombination rate of 4 cM/Mb (–rr cM_Mb:4,4,4,4,4), piRNA clusters at the end of each chromosome (–cluster kb:100,100,100,100,100), a transposition rate of 0.1 (–u 0.1), neutral TE insertions (–x 0.0), a population size of 1,000 (–N 1,000), and equal mobilization of FL and ID insertions (–fl_id 0.5). TE invasions were launched by introducing 250–300 randomly distributed FL insertions in the first population (frequency of f=1/2×N). The position and the frequency of the IDs (i.e., the ID fingerprint) were recorded in the output. We used a custom script to compute the genetic distance among simulated populations based on the ID fingerprints (*dm-simulations.py*). To mirror the treatment of samples from natural populations, we ignored IDs solely occurring in a single population. Trees were again generated with the bionj algorithm and the number of splits shared between the expected and observed trees were computed with the R package ape (comparePhylo; Paradis et al. 2004).

To obtain the expected number of shared splits under the null model, we simulated one million random trees with the same tips as the expected tree (rtree; Paradis et al. 2004). We then compared the distribution of shared splits from the random trees and the trees from 100 simulations. Shared splits were modeled as *binom*(*p*, *n*) with *n* equal to the maximum possible number of shared splits (in our case eight) and a flat prior distribution for *p*, with *beta*(1, 1). Posterior probability distributions were generated from four MCMC chains with 1,000 samples and a burn-in of 1,000 samples (R package greta; Golding 2019).

## Supplementary Material

Supplementary data are available at *Genome Biology and Evolution* online.

## Supplementary Material

evaa190_Supplementary_Data
