# Supplementary material for: Reconstructing the Invasion Route of the P-Element in Drosophila melanogaster Using Extant Population Samples
Source: Genome Biol Evol. 2020 Sep 10;12(11):2139–52. doi: 10.1093/gbe/evaa190 (PMC7750958; doi:10.1093/gbe/evaa190)
Supplement: evaa190_Supplementary_Data [file evaa190_supplementary_data.zip › supplement.pdf]

Supplementary Figures and Tables for: "Reconstructing the invasion route of the P-element in *Drosophila melanogaster* using extant population samples"

Lukas Weilguny, Christos Vlachos, Divya Selvaraju and Robert Kofler*

*correspondence to rokofler@gmail.com

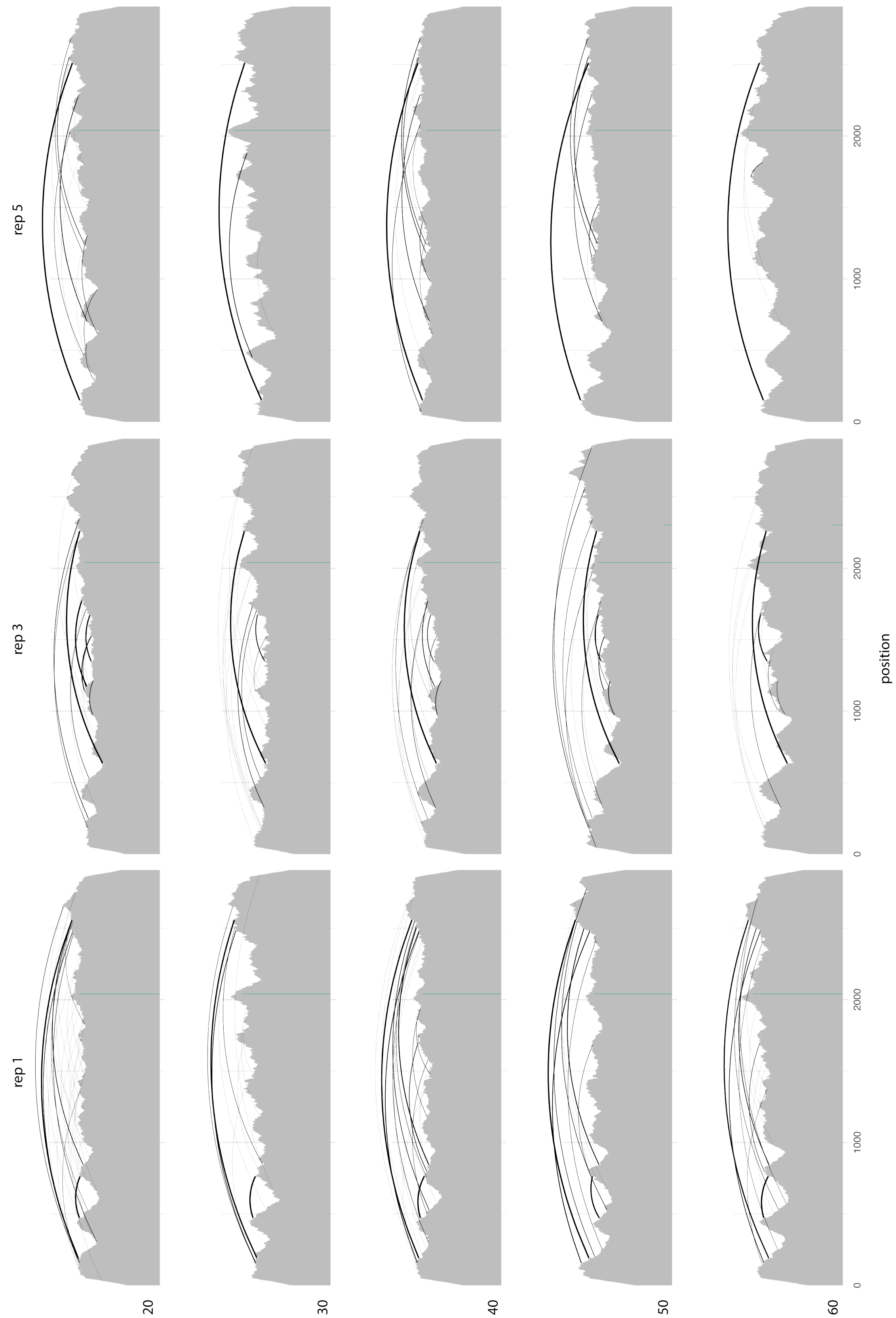

Figure S1: ID fingerprints of the P-element during an invasion in *D. simulans*. Results are shown for 3 replicates and generations 20-60. No noticeable ID fingerprints were found at earlier generations.

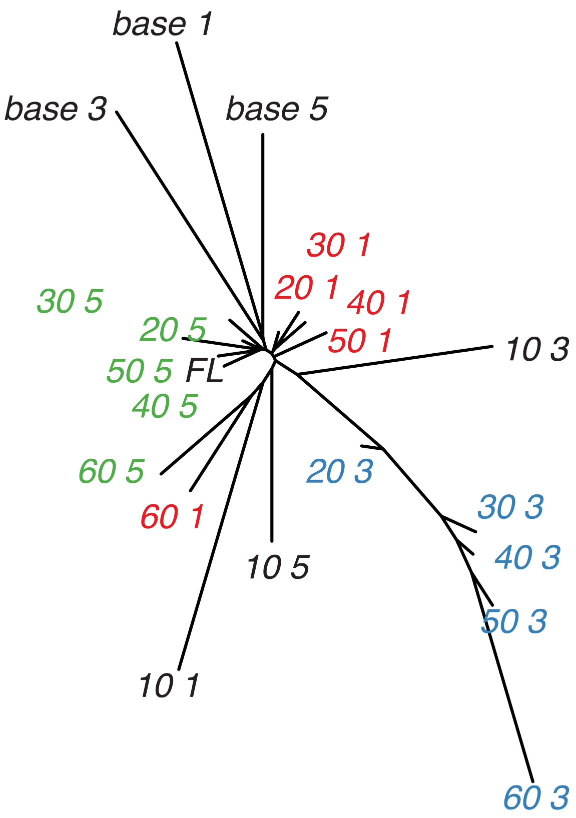

Figure S2: Unrooted tree constructed from SNPs within the P-element. A population solely containing consensus sequences of the P-element was used as outgroup (FL). The genetic distance was computed as average Jost's D among all SNPs.

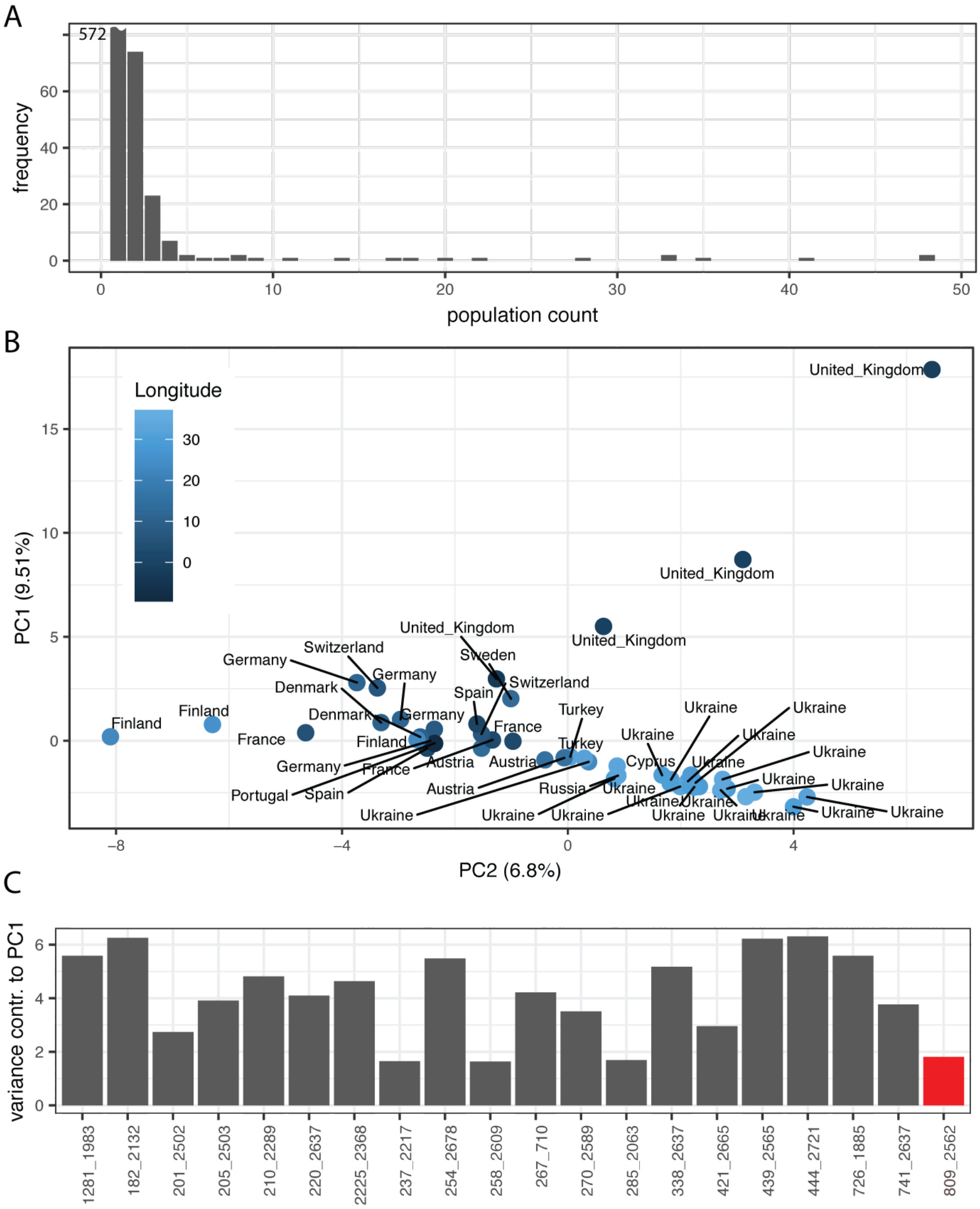

Figure S3: IDs of the P-element allows reproducing the longitudinal cline found in *D. melanogaster* populations from Europe (Kapun *et al.*, 2020). A) Distribution of IDs in the investigated populations. Most IDs (572) are only found in a single population while two are found in all 48 populations. B) Scaled PCA based on the frequency of IDs found in the populations. C) Variance contribution of the most important IDs to PC1. Labels on the x-axis indicate the start and end position of IDs. The KP-element is shown red.

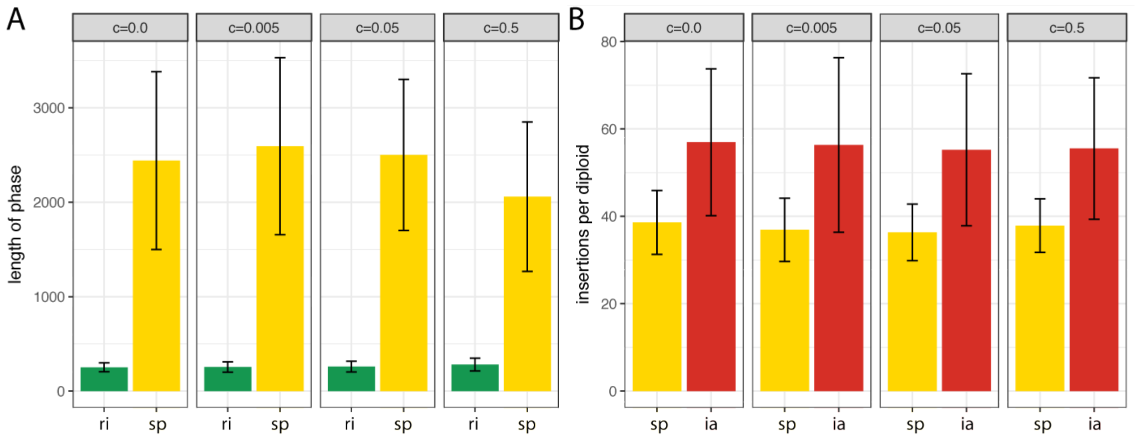

Figure S4: The probability that transposition of a FL element yields an ID (i.e. the conversion rate c) has little influence on the invasion dynamics. A) The conversion rate c has little influence on the length of a phase (Kruskal-Wallis rank sum test; $p_{ri} = 0.11$, $p_{sp} = 0.020$). B) The conversion rate c has little influence on the TE abundance at the beginning of a phase (Kruskal-Wallis rank sum test; $p_{sp} = 0.35$, $p_{ia} = 0.97$). ri: rapid invasion phase, sp: shotgun phase, ia: inactive phase

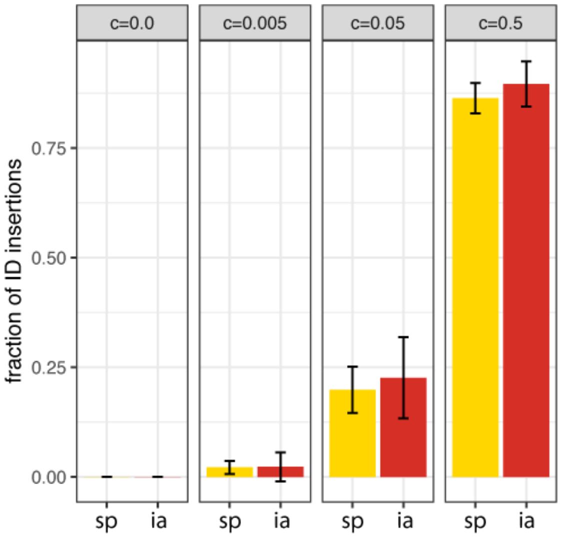

Figure S5: Once an invasion is silenced, the fraction of IDs remains fairly stable within populations. We measured the fraction of IDs at the onset of the shotgun phase (sp; silencing of TEs by segregating cluster insertions) and the inactive phase (ia; silencing of TEs by fixed cluster insertions). The difference in the fraction of IDs is small and mostly not significant (Wilcoxon rank sum test; $p_{c:0.005} = 0.15$, $p_{c:0.05} = 0.22$, $p_{c:0.5} = 0.0011$). sp shotgun phase, ia inactive phase

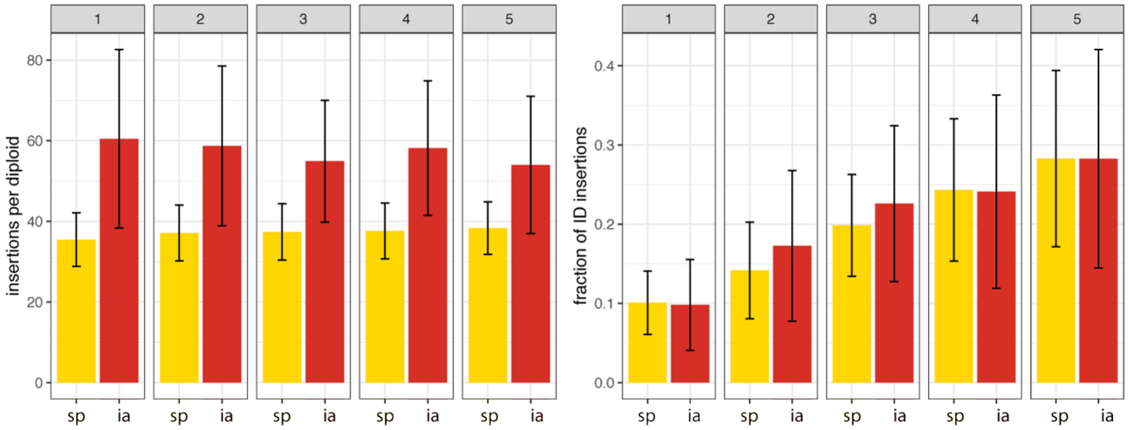

Figure S6: Although the TE abundance does not change in successively invaded populations, the fraction of TEs with an ID increases in successively invaded population. The top panel indicates the order in which the populations were invaded. A) The TE abundance, measured at the beginning of two phases, remains stable in successively invaded populations (Kruskal-Wallis rank sum test; $p_{sp} = 0.19$, $p_{ia} = 0.58$) B) The fraction of IDs, measured at the beginning of two phases, increases in successively invaded populations (Kruskal-Wallis rank sum test; $p_{sp} < 2.2e-16$, $p_{ia} = 1.5e-13$). sp shotgun phase, ia inactive phase

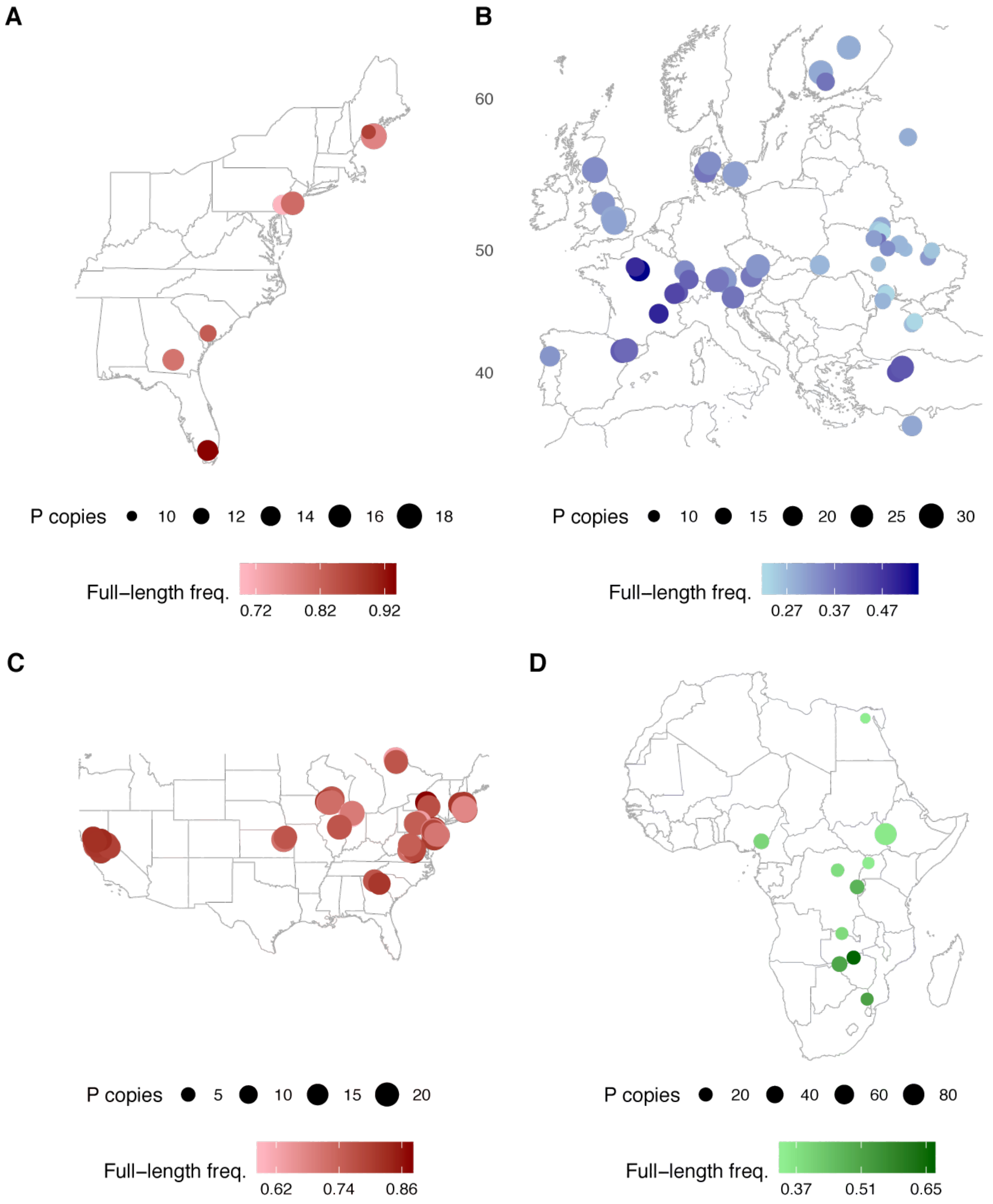

Figure S7: P-element abundance and abundance of FL elements in worldwide populations of *D. melanogaster*. Data are shown for four different data sets: A) East Coast of North America (Bergland *et al.*, 2014) B) Europe (Kapun *et al.*, 2020) C) North America (Machado *et al.*, 2018) and D) Africa (Lack *et al.*, 2015)

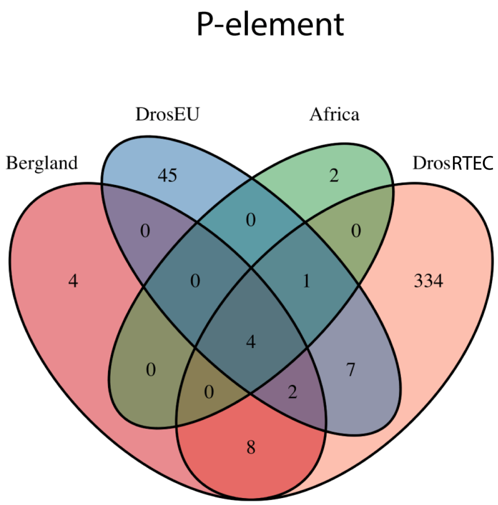

Figure S8: Prevalence of P-element IDs in different samples. Only IDs occurring in at least two populations were considered.

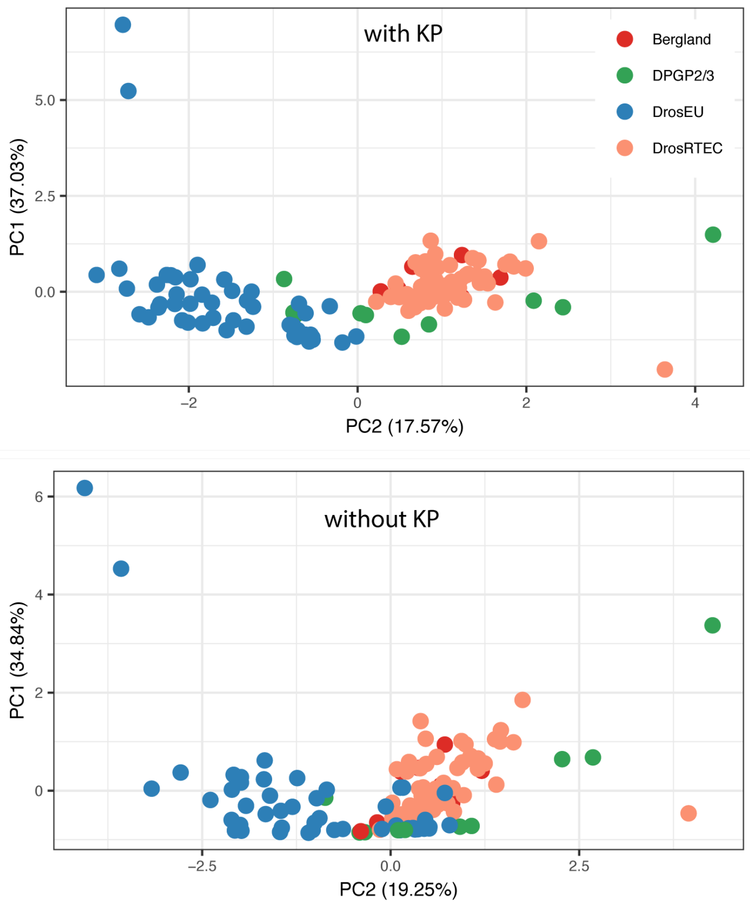

Figure S9: Scaled PCA based on the frequency of the six most abundant, world-wide IDs of the P-element. Note that most African samples (DPGP2/3) cluster between European (DrosEU) and North American samples (Bergland and DrosRTEC).

Table S1: Overview of the parameters used in simulations

N (population size)	1000
populations	10
u (transposition rate)	0.2
c (conversion rate)	0.025
duration of simulations	3000 generations
time interval between migration events	300 generations
migrating individuals per migration event	100
starting conditions	300 FL insertions randomly distributed in first population
additional assumptions	one ID per TE copy
	randomly picked breakpoints of IDs (positions $1 - 2907$)
	neutral TE insertions

Table S2: Overview of the short read data used in this work

publication	sample IDs
Kofler *et al.* (2018)	ERX2048594, ERX2048593, ERX2048592, ERX2048591, ERX2048590, ERX2048589, ERX2048588, ERX2048587, ERX2048586, ERX2048585, ERX2048584, ERX2048583, ERX2048582, ERX2048581, ERX2048580, ERX2048579, ERX2048578, ERX2048577, ERX2048576, ERX2048575, ERX2048574, ERX2048573, ERX2048572, ERX2048571, ERX2048570, ERX2048569, ERX2048568, ERX2048567, ERX2048566, ERX2048565, ERX2048564, ERX2048563, ERX2048562, ERX2048561, ERX2048560, ERX2048559
Bergland *et al.* (2014)	SRR1525685, SRR1525694, SRR1525695, SRR1525696, SRR1525698, SRR1525699, SRR1525768, SRR1525769
Kapun *et al.* (2020)	SRR5647729, SRR5647730, SRR5647731, SRR5647732, SRR5647733, SRR5647734, SRR5647735, SRR5647736, SRR5647737, SRR5647738, SRR5647739, SRR5647740, SRR5647741, SRR5647742, SRR5647743, SRR5647744, SRR5647745, SRR5647746, SRR5647747, SRR5647748, SRR5647749, SRR5647750, SRR5647751, SRR5647752, SRR5647753, SRR5647754, SRR5647755, SRR5647756, SRR5647757, SRR5647758, SRR5647759, SRR5647760, SRR5647761, SRR5647762, SRR5647763, SRR5647764, SRR5647765, SRR5647766, SRR5647767, SRR5647768, SRR5647769, SRR5647770, SRR5647771, SRR5647772, SRR5647773, SRR5647774, SRR5647775, SRR5647776
Machado *et al.* (2018)	SRR3590550, SRR3590551, SRR3590554, SRR3590555, SRR3590556, SRR3590557, SRR3590558, SRR3590559, SRR3590560, SRR3590561, SRR3590562, SRR3590563, SRR3939042, SRR3939043, SRR3939044, SRR3939045, SRR3939046, SRR3939047, SRR3939048, SRR3939049, SRR3939050, SRR3939051, SRR3939052, SRR3939054, SRR3939056, SRR3939057, SRR3939058, SRR3939059, SRR3939076, SRR3939077, SRR3939078, SRR3939080, SRR3939081, SRR3939082, SRR3939083, SRR3939084, SRR3939085, SRR3939086, SRR3939087, SRR3939088, SRR3939089, SRR3939091, SRR3939092, SRR3939093, SRR3939094, SRR3939095, SRR3939096, SRR3939097, SRR3939098, SRR3939099, SRR3939100, SRR3939101, SRR3939102, SRR3939103, SRR3939104
Lack *et al.* (2015)	SRR189040, SRR189045,SRR306633, SRR189277, SRR189279, SRR189281, SRR189389, SRR306609, SRR306611, SRR306612, SRR306614, SRR306616, SRR306618, SRR306619, SRR306621, SRR306622, SRR306623, SRR306624, SRR306629, SRR306630, SRR306631, SRR306632, SRR306634, SRR202089, SRR202123, SRR203068, SRR203069, SRR203226, SRR203232, SRR203233, SRR203330, SRR203336, SRR203473, SRR203474, SRR203496, SRR203502, SRR204007, SRR204018, SRR210794, SRR248129, SRR248130, SRR326790, SRR326796, SRR326800, SRR326933, SRR202128, SRR1686794, SRR1686796, SRR1686797, SRR1686964, SRR1688222

References

Bergland, A. O., Behrman, E. L., O'Brien, K. R., Schmidt, P. S., and Petrov, D. A. 2014. Genomic Evidence of Rapid and Stable Adaptive Oscillations over Seasonal Time Scales in Drosophila. *PLOS Genet.*, 10(11): e1004775.

Kapun, M., Barrón, M. G., Staubach, F., Vieira, J., Obbard, D. J., Goubert, C., Rota-Stabelli, O., Kankare, M., Haudry, A., Wiberg, R. A. W., *et al.* 2020. Genomic analysis of European *Drosophila melanogaster* populations on a dense spatial scale reveals longitudinal population structure and continent-wide selection. *Mol. Biol. Evol.*, page msaa120 (advance article).

Kofler, R., Senti, K.-A., Nolte, V., Tobler, R., and Schlötterer, C. 2018. Molecular dissection of a natural transposable element invasion. *Genome Res.*, 28(2): 824–835.

Lack, J. B., Cardeno, C. M., Crepeau, M. W., Taylor, W., Corbett-Detig, R. B., Stevens, K. A., Langley, C. H., and Pool, J. E. 2015. The Drosophila genome nexus: a population genomic resource of 623 *Drosophila melanogaster* genomes, including 197 from a single ancestral range population. *Genetics*, 199(4): 1229–41.

Machado, H., Bergland, A. O., Taylor, R., Tilk, S., Behrman, E., Dyer, K., Fabian, D., Flatt, T., Gonzalez, J., Karasov, T., Kozeretska, I., Lazzaro, B., Merritt, T., Pool, J., O'Brien, K., Rajpurohit, S., Roy, P., Schaeffer, S., Serga, S., Schmidt, P., and Petrov, D. 2018. Broad geographic sampling reveals predictable and pervasive seasonal adaptation in Drosophila. *bioRxiv*, page 337543.
