# Supplementary material for: Reconstructing the Invasion Route of the P-Element in Drosophila melanogaster Using Extant Population Samples
Source: Genome Biol Evol. 2020 Sep 10;12(11):2139–52. doi: 10.1093/gbe/evaa190 (PMC7750958; doi:10.1093/gbe/evaa190)
Supplement: evaa190_Supplementary_Data [file evaa190_supplementary_data.zip › Supplementary_text_1.pdf]

Supplementary text 1: Influence of different parameters on the accuracy of the inferred invasion routes

August 21, 2020

In this supplementary text we evaluate the influence of several parameters on the accuracy of the reconstructed invasion routes.

Sampling time

Genetic drift may distort ID fingerprints over time, we therefore asked how long invasion routes could be estimated. We inferred invasion routes at different time points after invasion of all 10 populations (≥ 3000 generations). Our simulations suggest that invasion routes may be traced for hundreds of generations after the spread of the TE, with only a small loss of accuracy over time (Fig. S1). However, in natural populations the accuracy of the inferred invasion routes may decline faster over time than in the simulations. For example, the deletion bias described for Drosophila (Petrov *et al.*, 1996) could continuously introduce novel IDs that blur invasion routes.

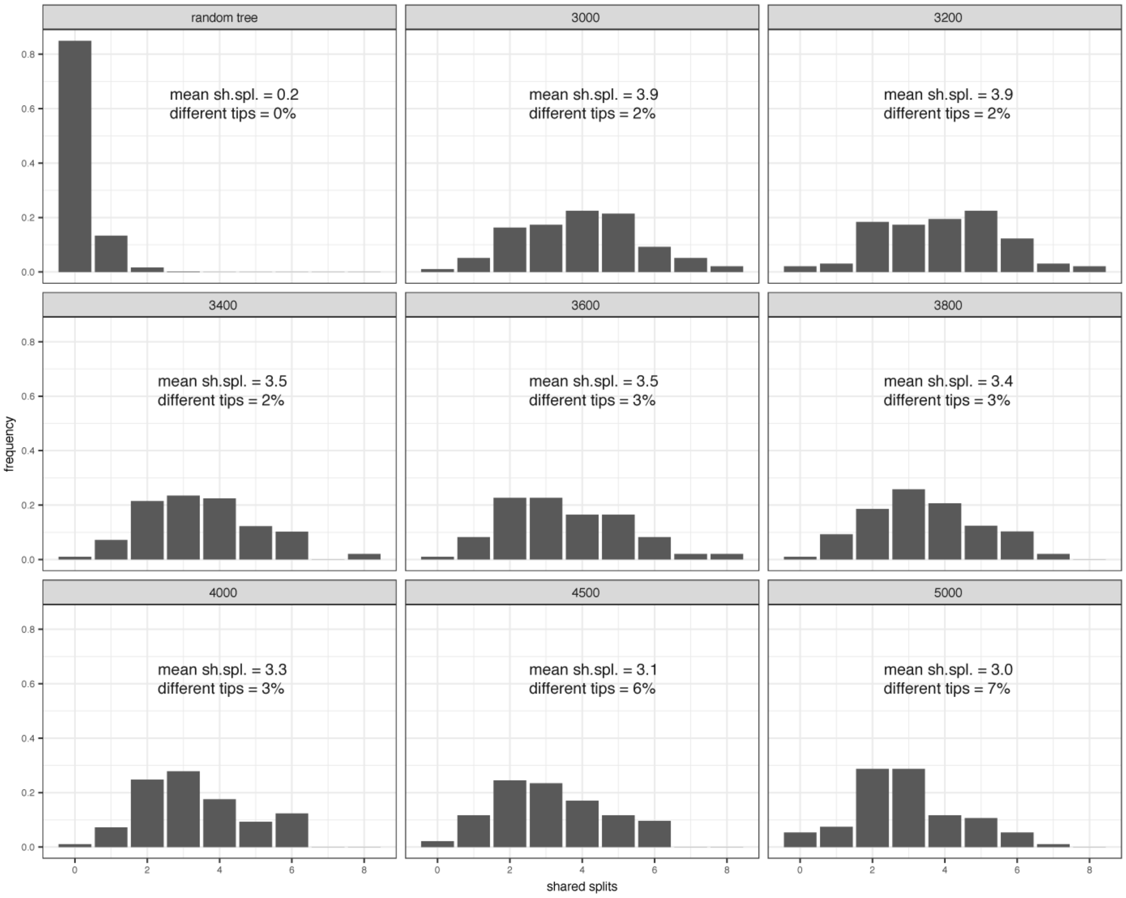

Figure S1: Influence of the sampling time (in generations; top panels) on the accuracy of the inferred invasion route. The accuracy is measured in shared splits (sh.spl.), where the maximal possible number of shared splits is 8. By generation 3000, all 10 simulated populations were invaded by the TE (it is not feasible to infer invasion routes prior to the invasion of all samples). We performed 100 simulations for each scenario, except for random trees, where 100,000 simulations were performed. The fraction of trees with different numbers of tips than expected is shown in the figure.

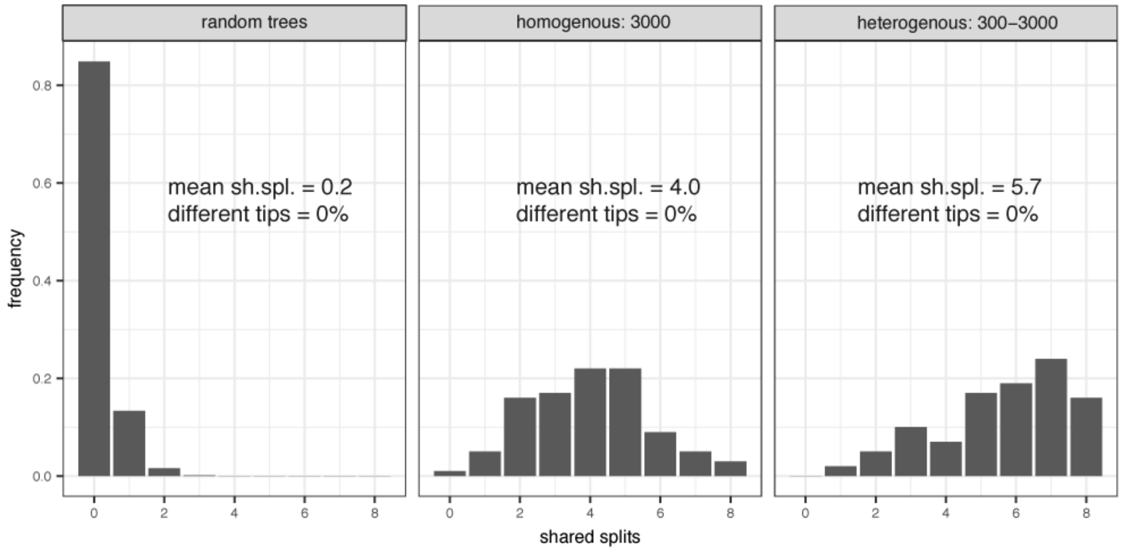

Figure S2: Influence of the heterogeneity of sampling time (in generations; top panels) on the accuracy of the inferred invasion route. We either sampled all populations at the same time (homogeneous 3000) or at different times (heterogeneous 300-3000) where the first population was sampled at generation 300, the second at generation 600, the third at generation 900, ..., and the tenth at generation 3000. By generation 3000, all 10 simulated populations were invaded by the TE. The accuracy is measured in shared splits (sh.spl.), with a maximal possible number of shared splits of 8. We performed 100 simulations for each scenario. Solely the random trees are based on 100,000 simulations. The fraction of trees with different numbers of tips than expected is shown in the figure.

Sampling heterogeneity

So far, we sampled all populations at the same time (e.g generation 3000). We further asked if other sampling strategies could enhance the accuracy of the inferred invasion routes. Especially sampling populations early may reduce the impact of drift and thus enhance the performance of our approach. To test this we sampled each population as early as possible, i.e. directly after a population was invaded (population 1 at generation 300, ... , population 10 at generation 3000). Note that this approach introduces substantial heterogeneity in the sampling time. Sampling populations early substantially improved the accuracy of our approach compared to sampling all populations at the same time at generation 3000 (1.6 more shared splits; Fig. S2). We thus conclude that it is more important to sample populations early than at the same time.

Test statisitc

Based on our simulations we evaluated the performance of different distance metrics for estimating the similarity of ID fingerprints among populations. The difference in the fraction of FL insertions had the worst performance (shared splits = 2.4), followed by Jost's D (shared splits = 3.4; Fig. S3). Removing IDs solely found in a single population significantly increased the accuracy of Jost's D (mean shared splits = 4.0; Wilcoxon rank sum test $p = 0.013$; Fig. S3). Finally, the inverse of the number of IDs shared between two populations ($1/shared$) performed best (shared splits = 5.3; Fig. S3). This approach may, however, not be suitable for real data as the number of shared IDs will be highly susceptible to differences in sampling and sequencing depth among samples (in our simulations all IDs are known).

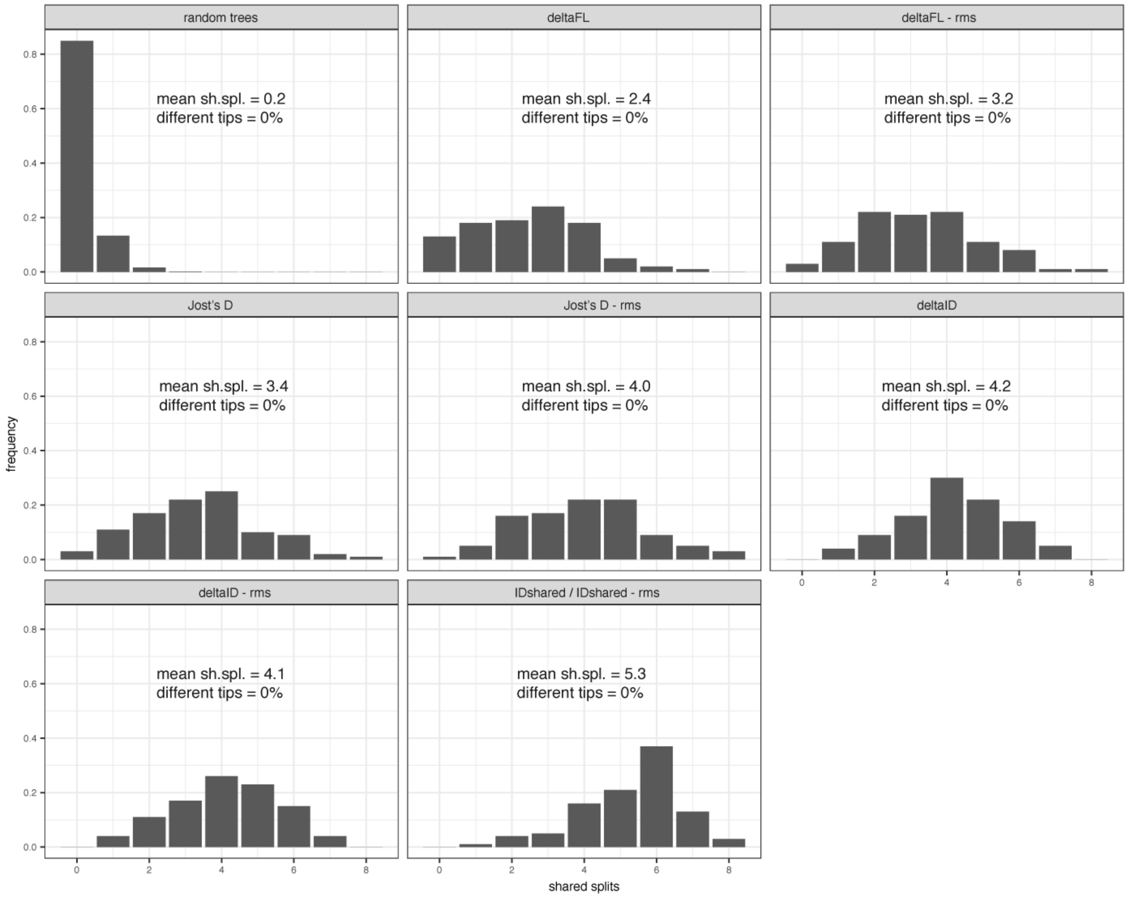

Figure S3: Influence of the test statistic on the accuracy of the inferred invasion route. The accuracy is measured in shared splits (sh.spl.), with a maximal possible number of shared splits of 8. Each test statistic was evaluated with 100 simulations and 100,000 simulations were performed for the random trees. The fraction of trees with different numbers of tips than expected is shown in the figure. deltaFL, difference in the fraction of FL insertions between two populations; deltaID, sum of ID frequency differences between two populations; IDshared, inverse of the number of IDs shared between two populations; rms, remove IDs solely occurring in a single population

Conversion rate

The conversion rate c is a crucial parameter. If the conversion rate is low, few IDs will emerge and tracing the invasion will not be feasible. Our simulations suggest that conversion rates $c > 0.01$ are necessary to trace invasions with reasonable accuracy (Fig. S4). During a P-element invasion in *D. simulans*, the fraction of IDs in populations plateaued at about 13% (Kofler *et al.*, 2018). This roughly corresponds to a conversion rate of $c \approx 0.03$ (interpolating the plateauing level from our simulations; main manuscript, Fig. 5). Thus, the conversion rate of the P-element, at least in *D. simulans*, is likely within the parameter range resulting in accurate invasion paths (Fig. S4).

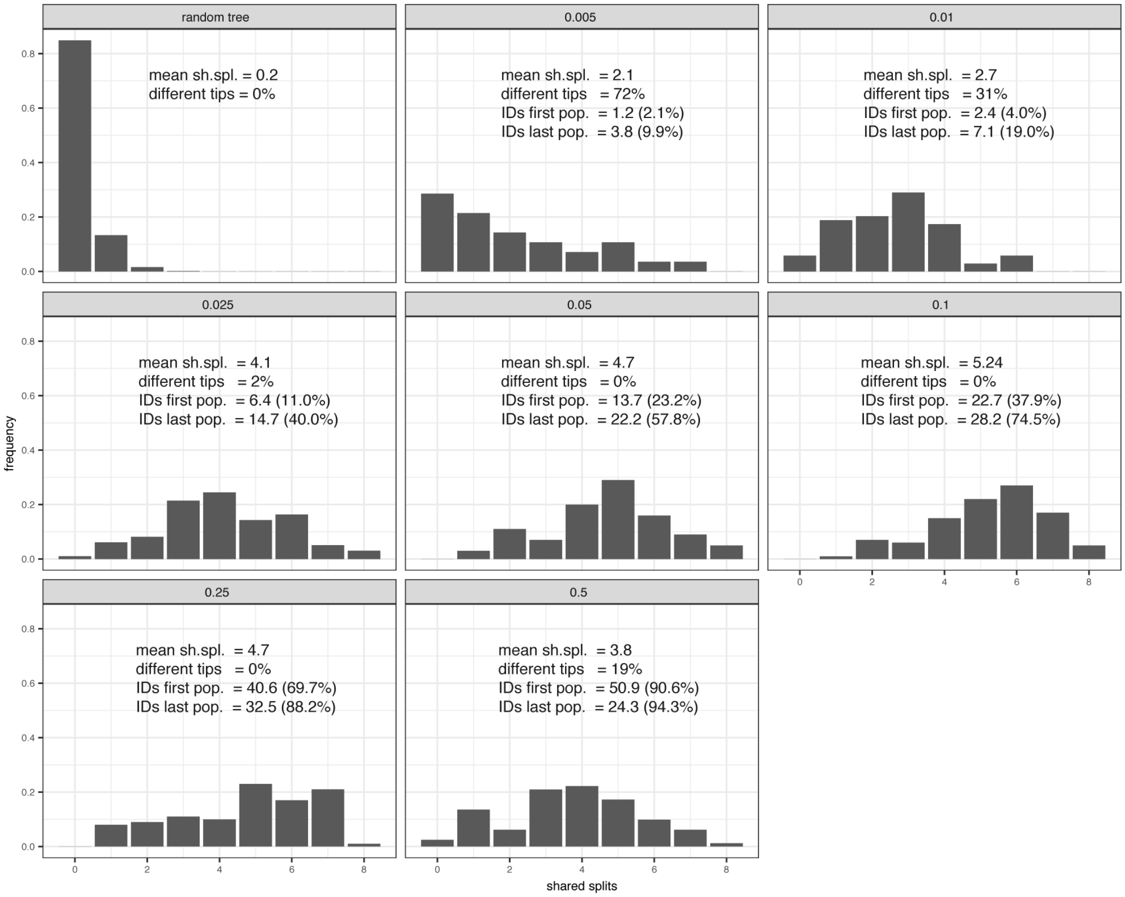

Figure S4: Influence of the conversion rate (c; top panel) on the accuracy of the inferred invasion route. The accuracy is measured in shared splits (sh.spl.), where the maximal possible number of shared splits is 8. We performed 100 simulations for each scenario, except for the random trees, where 100,000 simulations were performed. The fraction of trees having different numbers of tips than the expected one is shown in the figure. The average number of ID TEs per diploid and the fraction of TEs with an ID is shown for the first and the last of the 10 simulated populations (IDs first pop. and IDs last pop.)

TE abundance (size of piRNA clusters)

We were also interested in the influence of the TE abundance on the accuracy of our approach. However, in our model we do not control the TE abundance directly, rather the TE abundance is an outcome of the size of piRNA clusters (Kofler, 2019; Kelleher *et al.*, 2018). Hence the influence of the TE abundance may be investigated by modulating the size of piRNA clusters. As expected, the accuracy of our approach increases dramatically with TE abundance (Fig. S5). With about 27 - 59 TE copies per diploid, as for example found for most TE families in *D. melanogaster* (Kofler, 2019), our approach has a good accuracy (4-3 shared splits; Fig. S5).

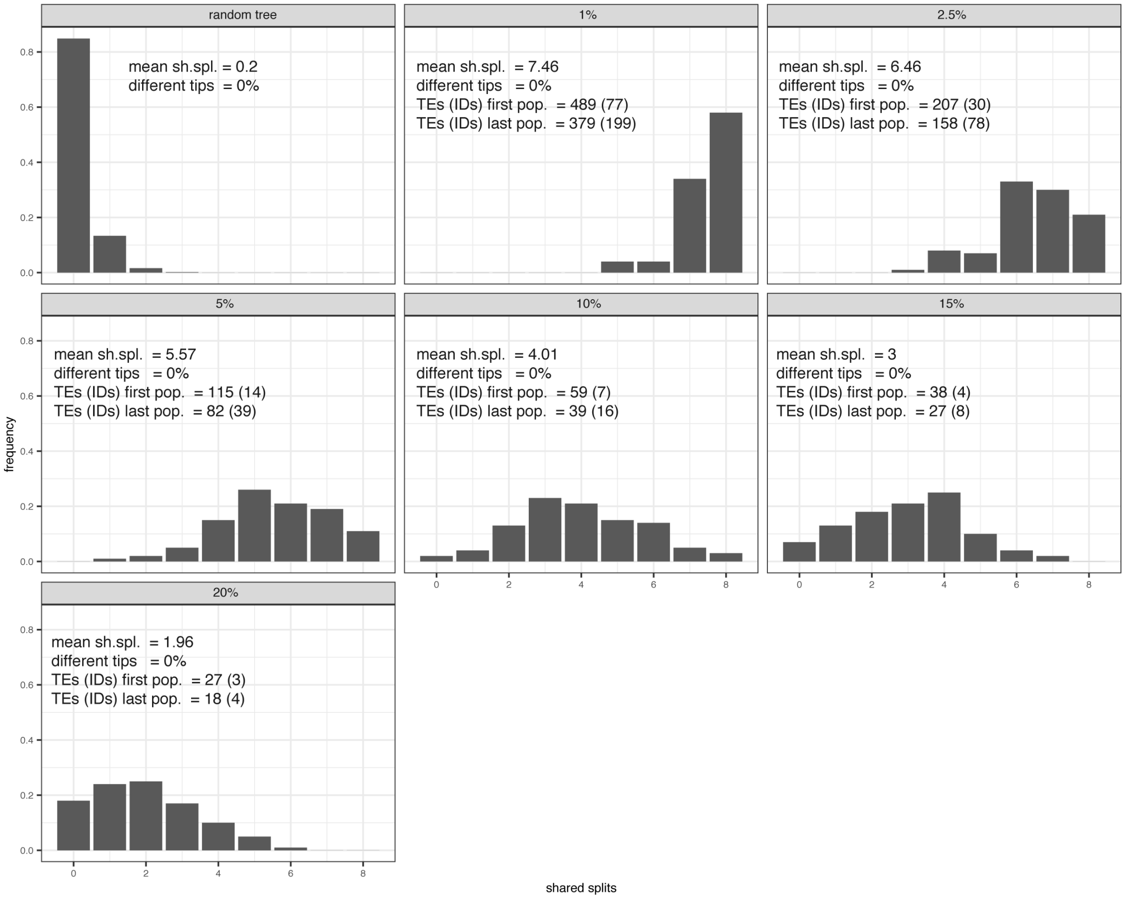

Figure S5: Influence of the size of piRNA clusters (top panel) and thus the TE abundance on the accuracy of the inferred invasion route. The accuracy is measured in shared splits (sh.spl.), where the maximal possible number of shared splits is 8. We performed 100 simulations for each scenario, except for the random trees, where 100,000 simulations were performed. The fraction of trees having different numbers of tips than the expected one is shown in the figure. Furthermore we show the number of TEs and IDs per diploid in the first and last population.

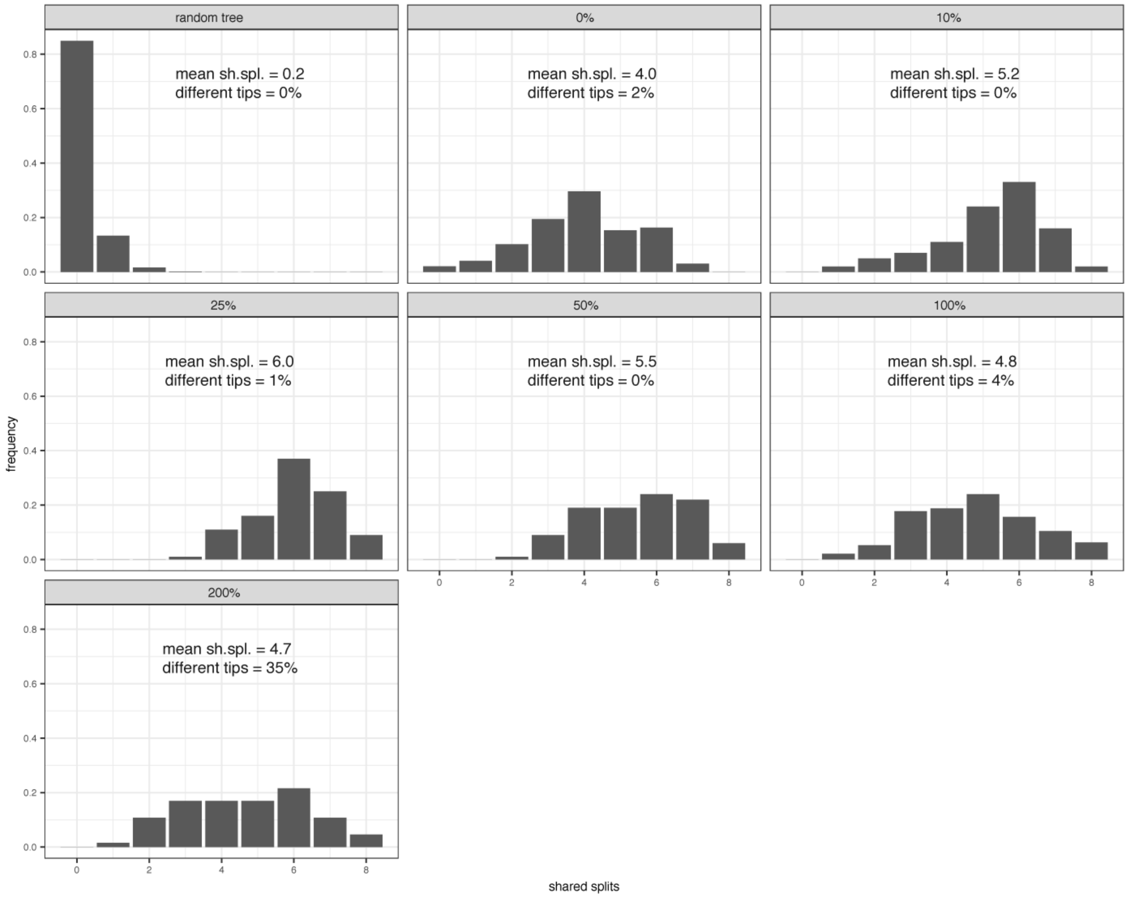

Figure S6: Influence of preferential mobilization of ID elements (in percent elevated transposition rate of ID over FL elements; top panel) on the accuracy of the inferred invasion route. The accuracy is measured in shared splits (sh.spl.), where the maximal possible number of shared splits is 8. We performed 100 simulations for each scenario, except for the random trees, where 100,000 simulations were performed. The fraction of trees having different numbers of tips than the expected one is shown in the figure.

Preferential mobilization of IDs

In our model we assumed that both FL and ID elements are mobilized at the same rate (i.e. in the presence of an autonomous FL element). It was, however, suggested that IDs may be mobilized at a higher rate than FL elements (Itoh *et al.*, 2007; Kofler *et al.*, 2018). We thus performed simulations with different transposition rates for FL and ID elements. Preferential mobilization of IDs greatly enhances the accuracy of our approach (Fig. S6). For example, if IDs are 25% more readily mobilized than FL elements, the accuracy of the reconstructed invasion route increased by 50% (Fig. S6).

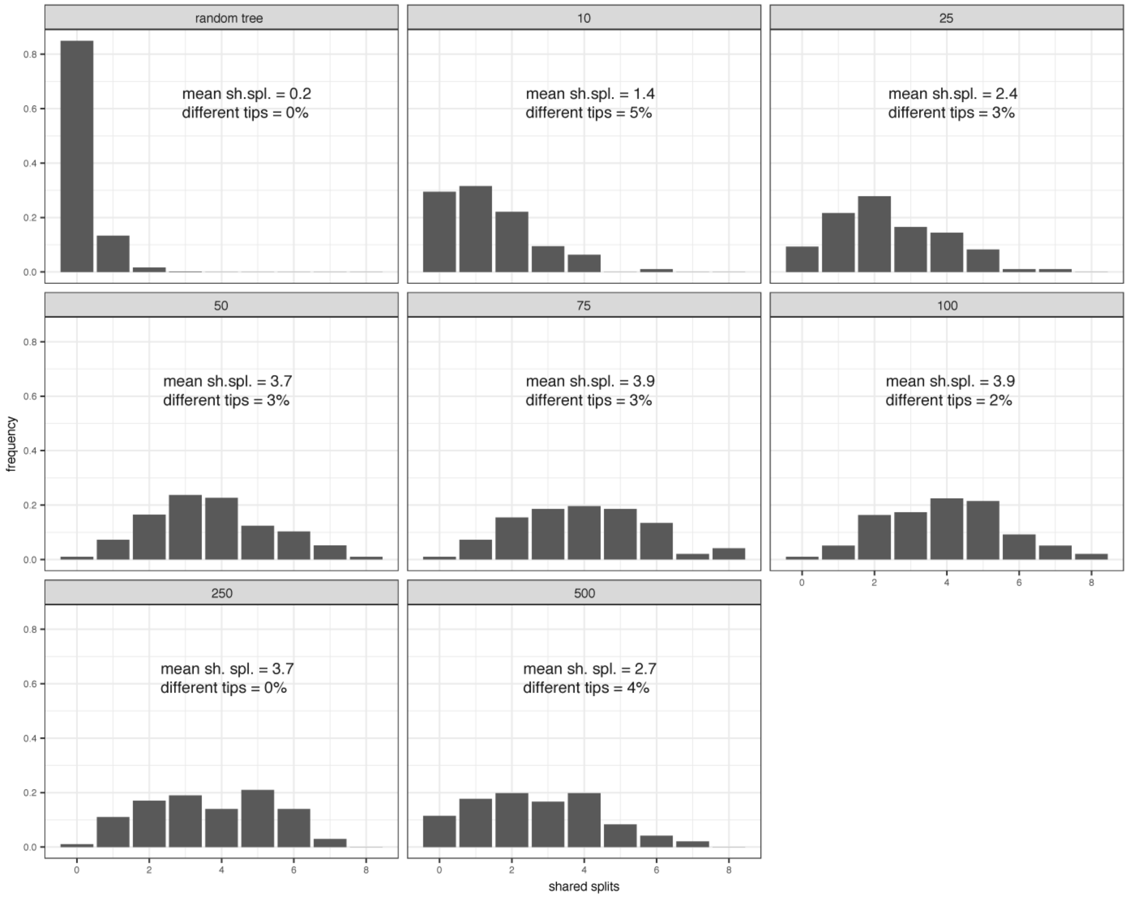

Figure S7: Influence of the number of migrants (top panel) on the accuracy of the inferred invasion route. The accuracy is measured in shared splits (sh.spl.), where the maximal possible number of shared splits is 8. We performed 100 simulations for each scenario, except for the random trees, where 100,000 simulations were performed. The fraction of trees having different numbers of tips than the expected one is shown in the figure.

Number of migrating individuals

The migration rate also influences the accuracy of our approach. If the number of migrants is small, few IDs will be carried to the target population, and thus the similarity of ID fingerprints among populations will be too low to reconstruct invasion routes. In our simulations about 50 migrants were necessary to trace invasions with reasonable accuracy (Fig. S7). Interestingly, larger numbers of migrants (> 50) only had a minor effect on the accuracy of our approach (Fig. S7).

Migration pattern

The migration pattern also influences the accuracy of our approach. Initially, we simulated unidirectional and unique migration events from an invaded source population into a naive target population, thus triggering a novel invasion in the target population. With this approach we ensured that the inferred invasion route is solely based on the migration events that triggered the invasion and not on migration events following the invasion. However, in natural populations migration between neighboring populations will likely occur more regularly and in both directions. Interestingly, bidirectional and recurrent migration (100 migrants in both directions at each 300th generation) increased the accuracy of our approach by 41% (from 3.9 to 5.5 shared splits at generation 3000; Fig. S8). Furthermore, invasions could be traced with a high precision for hundreds of generations after the spread of the TE (> 3000 generation; Fig. S8). Under this model, assuming stable migration routes, recurrent migration likely increases the similarity of ID fingerprints between neighboring populations, which facilitates reconstructing the invasion route. However, if migration routes change over time recurrent migration will decrease the accuracy of our approach.

So far we introduced migration events at each 300th generation. With this approach we allowed TEs to completely invade a population before introducing the next migration event from the most recently invaded population to the next naive population. Within these 300 generations, TEs will mostly be silenced by segregating piRNA cluster insertions and populations will have acquired fairly stable ID fingerprints. It may, however, be argued that such a long pause between migration events is an implausible model for natural populations. For natural TE invasions the time required to silence a TE could actually be substantially shorter. For example, the P-element invasion in experimental *D. simulans* populations was silenced within a mere 20 generations (Kofler *et al.*, 2018). The reason for this discrepancy between real and simulated data, is however not clear (the transposition rate was similar: $u = 0.1$). We speculate that an insertion bias into piRNA clusters, as described for the P-element, could accelerate silencing of the TE (Kofler *et al.*, 2018; Karpen and Spradling, 1992; Zhang *et al.*, 2020). Paramutations, which convert euchromatic TE insertions into piRNA producing loci could also speed up silencing of an invasion (de Vanssay *et al.*, 2012; Mohn *et al.*, 2014). Hence, natural populations may acquire stable ID fingerprints much faster than our simulated populations. Nevertheless, we found that our approach also works when populations do not have sufficient time to establish stable ID fingerprints. With a more classic migration model, i.e single bidirectional and recurrent migrant between neighboring populations at each generation, the invasion route can still be inferred, albeit with a reduced accuracy (Fig. S9).

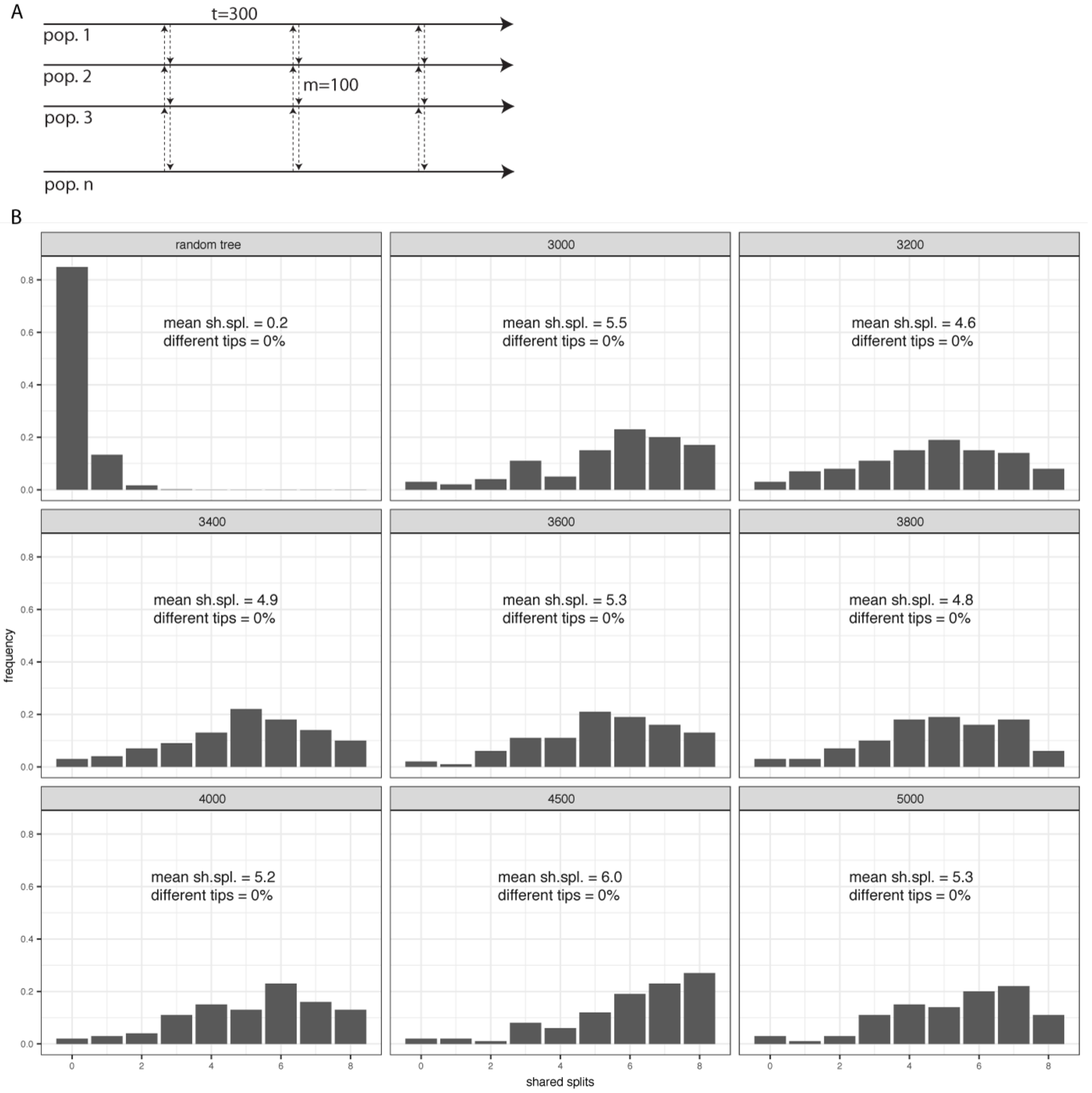

Figure S8: Influence of recurrent and bidirectional migration on the accuracy of the inferred invasion route. A) We simulated bidirectional and recurrent migration every 300th generation between neighboring populations (with $m = 100$ migrants). B) Accuracy of the inferred invasion history in shared splits (sh.spl.) at different time points (in generations, top panel). Note that by generation 3000 all 10 simulated populations were invaded (it is not feasible to infer invasion routes prior to the invasion of all samples). The maximal possible number of shared splits is 8. We performed 100 simulations for each scenario, except for the random trees, where 100,000 simulations were performed. The fraction of trees with different numbers of tips than expected is shown in the figure. Compared to unidirectional-unique migration (Fig. S1), bidirectional-recurrent migration increased the accuracy of our approach from 3.9 to 5.5 shared splits (at generation 3000).

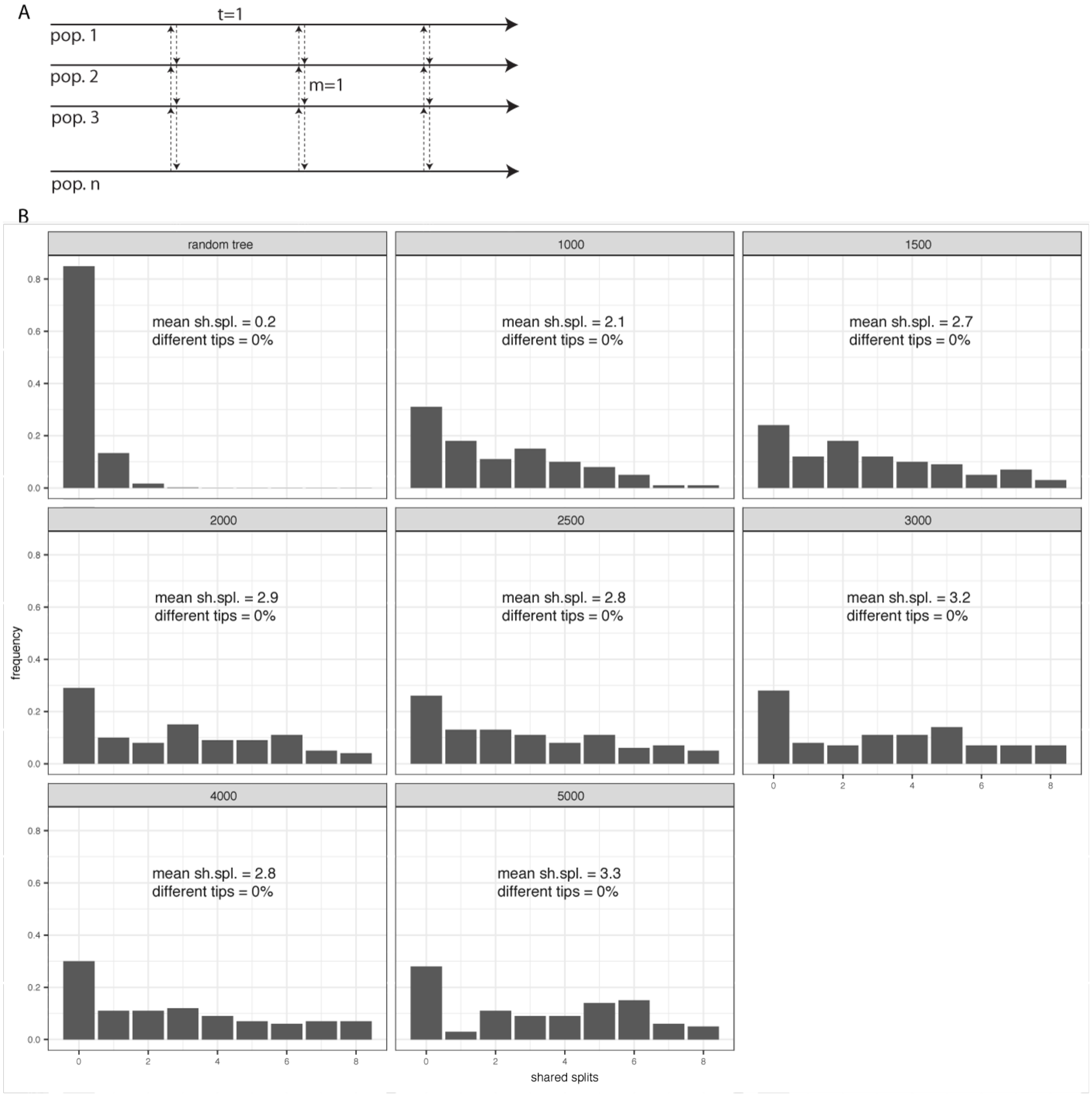

Figure S9: Influence of recurrent and bidirectional migration on the accuracy of the inferred invasion history of a TE. A) We simulated bidirectional migration of $m = 1$ migrant at each generation ($t = 1$) between neighboring populations. B) Accuracy of the inferred invasion history in shared splits (sh.spl.) at different time points (in generations, top panel). The maximal possible number of shared splits is 8. We performed 100 simulations for each scenario, except for the random trees, where 100,000 simulations were performed. The fraction of trees having different numbers of tips than the expected one is shown in the figure.

References

de Vanssay, A., Bougé, A.-L., Boivin, A., Hermant, C., Teysset, L., Delmarre, V., Antoniewski, C., and Ronsseray, S. 2012. Paramutation in Drosophila linked to emergence of a piRNA-producing locus. *Nature*, 490(7418): 112–115.

Itoh, M., Takeuchi, N., Yamaguchi, M., Yamamoto, M. T., and Boussy, I. A. 2007. Prevalence of full-size P and KP elements in North American populations of *Drosophila melanogaster*. *Genetica*, 131(1): 21–28.

Karpen, G. H. and Spradling, A. C. 1992. Analysis of subtelomeric heterochromatin in the Drosophila minichromosome Dp1187 by single P element insertional mutagenesis. *Genetics*, 132(3): 737–753.

Kelleher, E. S., Azevedo, R. B. R., and Zheng, Y. 2018. The Evolution of Small-RNA-Mediated Silencing of an Invading Transposable Element. *Genome Biol. Evol.*, 10(11): 3038–3057.

Kofler, R. 2019. Dynamics of transposable element invasions with piRNA clusters. *Mol. Biol. Evol.*, 36(7): 1457–1472.

Kofler, R., Senti, K.-A., Nolte, V., Tobler, R., and Schlötterer, C. 2018. Molecular dissection of a natural transposable element invasion. *Genome Res.*, 28(2): 824–835.

Mohn, F., Sienski, G., Handler, D., and Brennecke, J. 2014. The rhino-deadlock-cutoff complex licenses noncanonical transcription of dual-strand piRNA clusters in Drosophila. *Cell*, 157(6): 1364–1379.

Petrov, D. A., Lozovskaya, E. R., and Hartl, D. L. 1996. High intrinsic rate of DNA loss in *Drosophila*. *Nature*, 384(6607): 346–9.

Zhang, S., Pointer, B., and Kelleher, E. S. 2020. Rapid evolution of piRNA-mediated silencing of an invading transposable element was driven by abundant de novo mutations. *Genome Res.*, 30(4): 566–575.
