# Supplementary material for: Reconstructing the Invasion Route of the P-Element in Drosophila melanogaster Using Extant Population Samples
Source: Genome Biol Evol. 2020 Sep 10;12(11):2139–52. doi: 10.1093/gbe/evaa190 (PMC7750958; doi:10.1093/gbe/evaa190)
Supplement: evaa190_Supplementary_Data [file evaa190_supplementary_data.zip › Supplementary_text_2.pdf]

Supplementary text 2: Reconstructing the invasion route of hobo

August 21, 2020

We aimed to investigate whether our approach allows us to shed light on the invasion route of a TE with a controversial invasion history, i.e. hobo in *D. melanogaster* (Fig. S1). Similarly to the P-element, hobo invaded worldwide *D. melanogaster* populations within the last 100 years (Daniels *et al.*, 1990; Pascua and Periquet, 1991). The hobo invasion likely predates the P-element invasion (Periquet *et al.*, 1989; Pascua and Periquet, 1991). Many strains collected in North America before 1955 contained hobo sequences, whereas few of the European strains collected before 1955 do (Periquet *et al.*, 1989). It was thus suggested that the hobo invasion started in North America (Periquet *et al.*, 1989). Alternatively, Europe could be the origin of the hobo invasion since the diversity of internal tandem repeats is highest in European populations (Bonnivard *et al.*, 2000). The high divergence between the canonical hobo sequence and hobo sequences of Drosophila species from Neotropical regions also suggests an origin of hobo in the Old World (Bernardo and Loreto, 2013). Finally, populations from Kenya showed the highest hobo activity, raising the possibility that hobo spread from Africa to other continents (Bonnivard *et al.*, 2000).

In addition to hobo insertions with a high similarity to the consensus sequence of hobo, most *D. melanogaster* strains also harbour fragmented and highly diverged hobo sequences (Fig. S2 (Pascua and Periquet, 1991; Periquet *et al.*, 1989)). It was thus suggested that hobo invaded *D. melanogaster* populations in two waves (Daniels *et al.*, 1990; Blumenstiel, 2019). To avoid confounding IDs from different invasion waves we restricted our analysis to reads with ≤ 1 mismatch with the hobo consensus sequence (i.e allowing for 1% divergence; the invasion route with all reads can be found in Fig. S3).

Based on our approach, we suggest that hobo invaded populations from Africa first, then spread to North America and finally invaded European populations (Fig. S1). Similarly to the P-element, populations from Ukraine were invaded last (Fig. S1). The route inferred by our approach is thus in agreement with the proposed invasion of North American populations prior to European ones (Periquet *et al.*, 1989) and an origin of the hobo invasion in Africa (Bonnivard *et al.*, 2000) (or in the Old World (Bernardo and Loreto, 2013)), but not with an origin of the invasion in Europe (Bonnivard *et al.*, 2000).

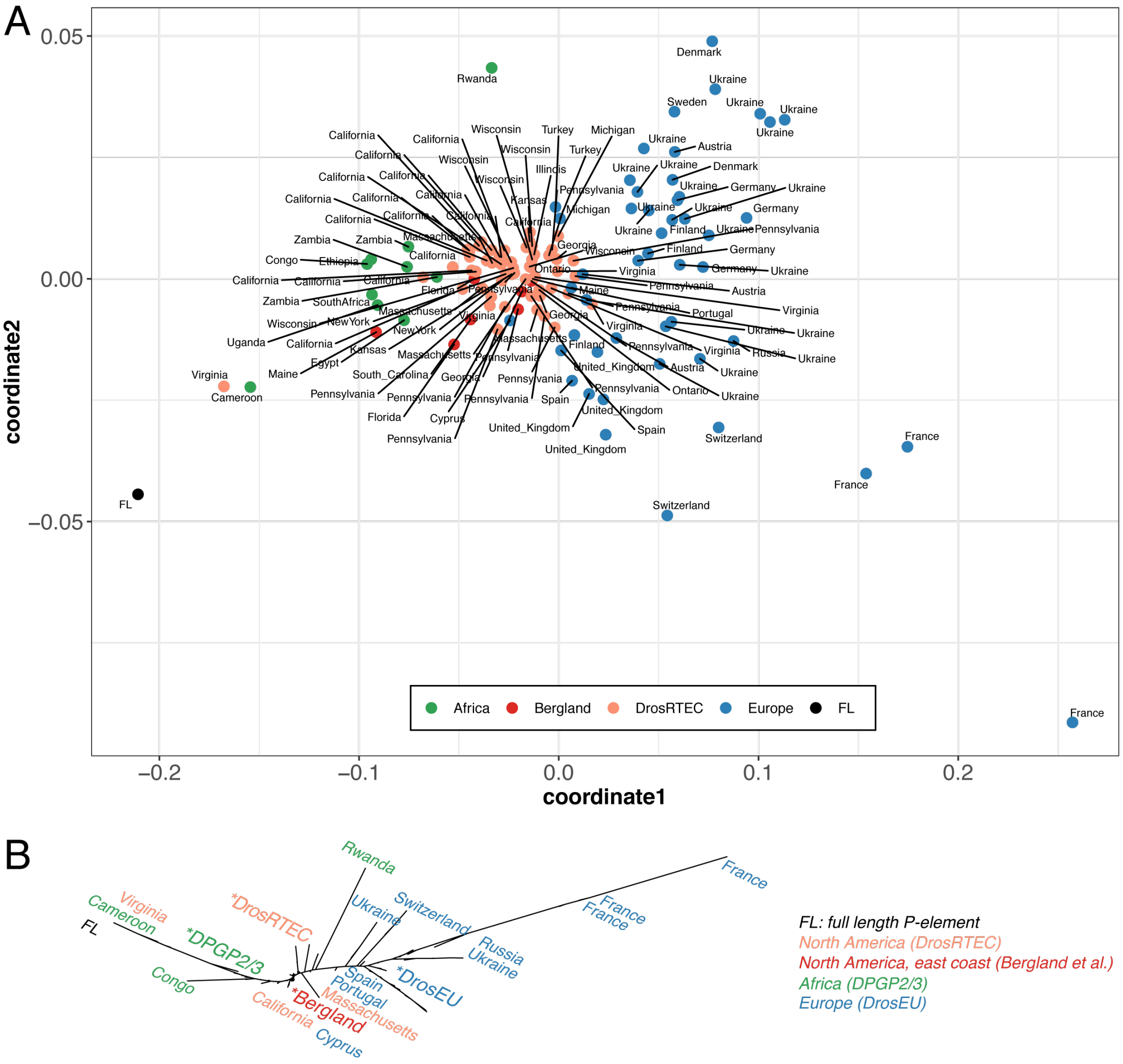

Figure S1: Invasion route of the hobo element in worldwide populations of *D. melanogaster*. A) The MDS plot is based on the similarity of ID fingerprints among populations. B) Tree showing the invasion route of hobo. Hobo invaded African populations first, subsequently spread to North America and finally invaded European populations. ∗ collapsed overlapping samples

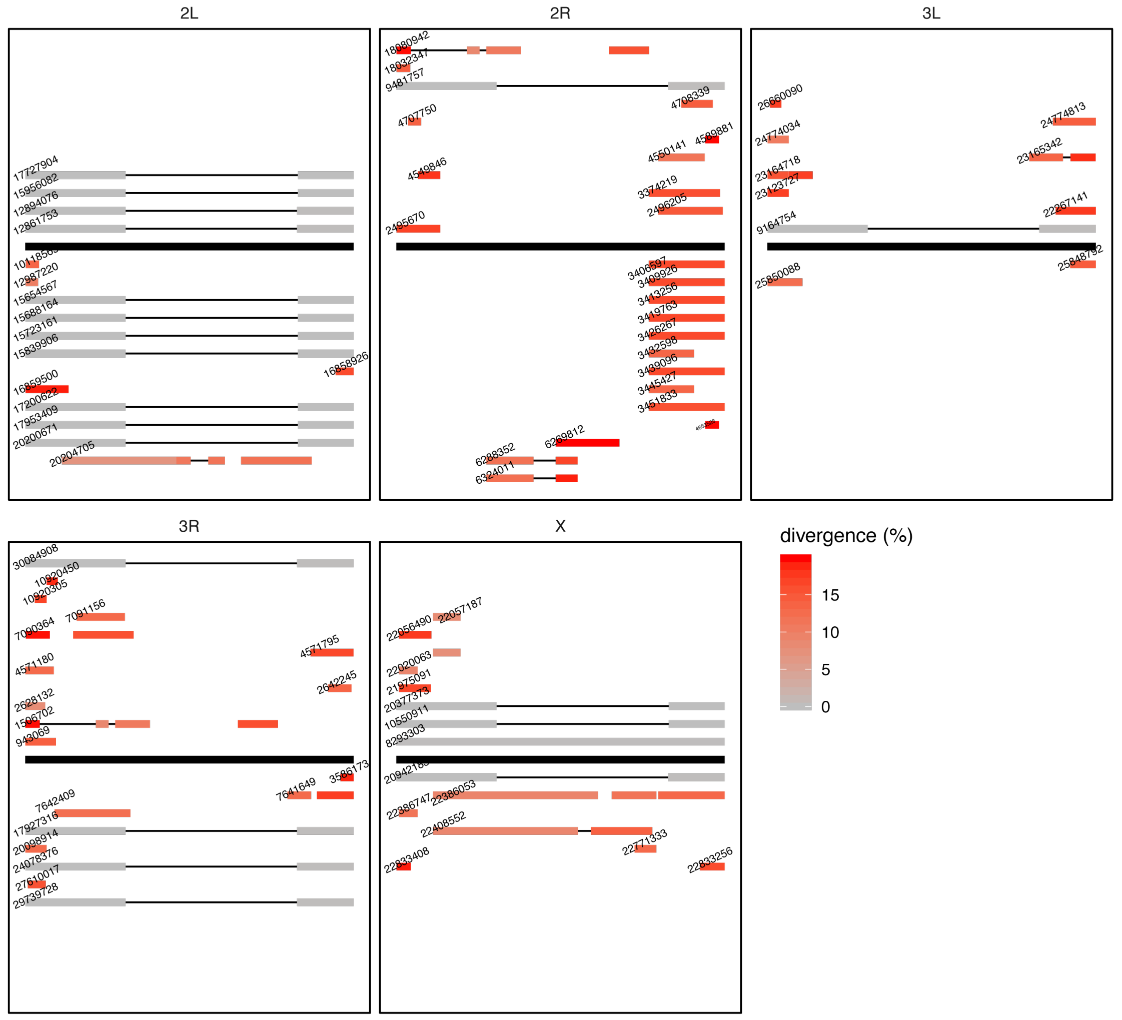

Figure S2: Overview of hobo insertions found in the *D. melanogaster* genome (release 6). For each hobo insertion we show an alignment with the hobo consensus sequence. IDs are indicated as lines and divergence to the consensus sequence is shown in color. Results are shown for all chromosomes separately (text indicates genomic position). Multiple IDs within an insertion are solely found for two highly diverged copies.

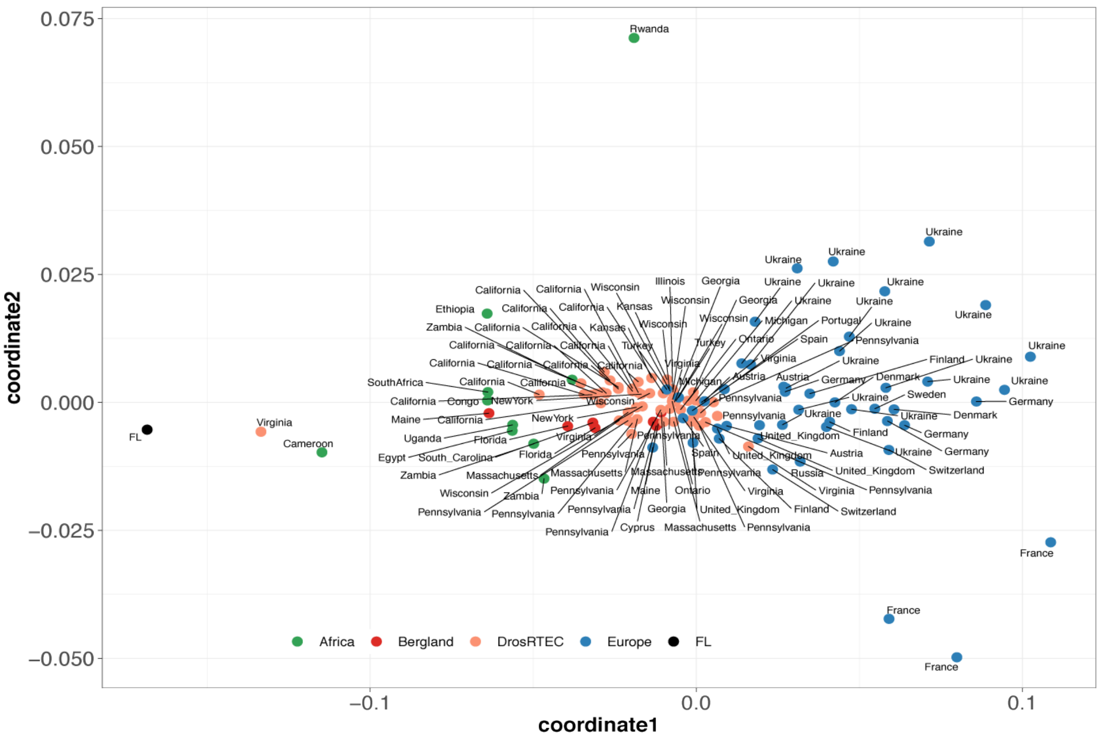

Figure S3: Invasion route of the hobo element in worldwide populations of *D. melanogaster* using all reads (≥ 0 mismatches). The MDS plot is based on the similarity of ID fingerprints among populations. We suggest that hobo invaded African populations first, then spread to North America and finally invaded European populations.

References

Bernardo, L. P. and Loreto, E. L. S. 2013. Hobo-Brothers Elements and Their Time and Place for Horizontal Transfer. *Genetica*, 141(10-12): 471–478.

Blumenstiel, J. P. 2019. Birth, School, Work, Death and Resurrection: The Life Stages and Dynamics of Transposable Element Proliferation. *Genes*, 10(5): 336.

Bonnivard, E., Bazin, C., Denis, B., and Higuet, D. 2000. A scenario for the hobo transposable element invasion, deduced from the structure of natural populations of *Drosophila melanogaster* using tandem TPE repeats. *Genet. Res. (Camb.)*, 75(1): 13–23.

Daniels, S. B., Chovnick, A., and Boussyy, I. A. 1990. Distribution of hobo transposable elements in the genus Drosophila. *Mol. Biol. Evol.*, 7(6): 589–606.

Pascua, L. and Periquet, G. 1991. Distribution of hobo transposable elements in natural populations of *Drosophila melanogaster. Mol. Biol. Evol.*, 8(3): 282–296.

Periquet, G., Hamelin, M. H., Bigot, Y., and Lepissier, A. 1989. Geographical and historical patterns of distribution of hobo elements in *Drosophila melanogaster* populations. *J. Evol. Biol*, 2(3): 223–229.
