# Supplementary material for: Reconstructing the Invasion Route of the P-Element in Drosophila melanogaster Using Extant Population Samples
Source: Genome Biol Evol. 2020 Sep 10;12(11):2139–52. doi: 10.1093/gbe/evaa190 (PMC7750958; doi:10.1093/gbe/evaa190)
Supplement: evaa190_Supplementary_Data [file evaa190_supplementary_data.zip › HistoryOfPInvasion_forGBE.zip!/HistoryOfPInvasion/figures_vector/fig1_v2.pdf]

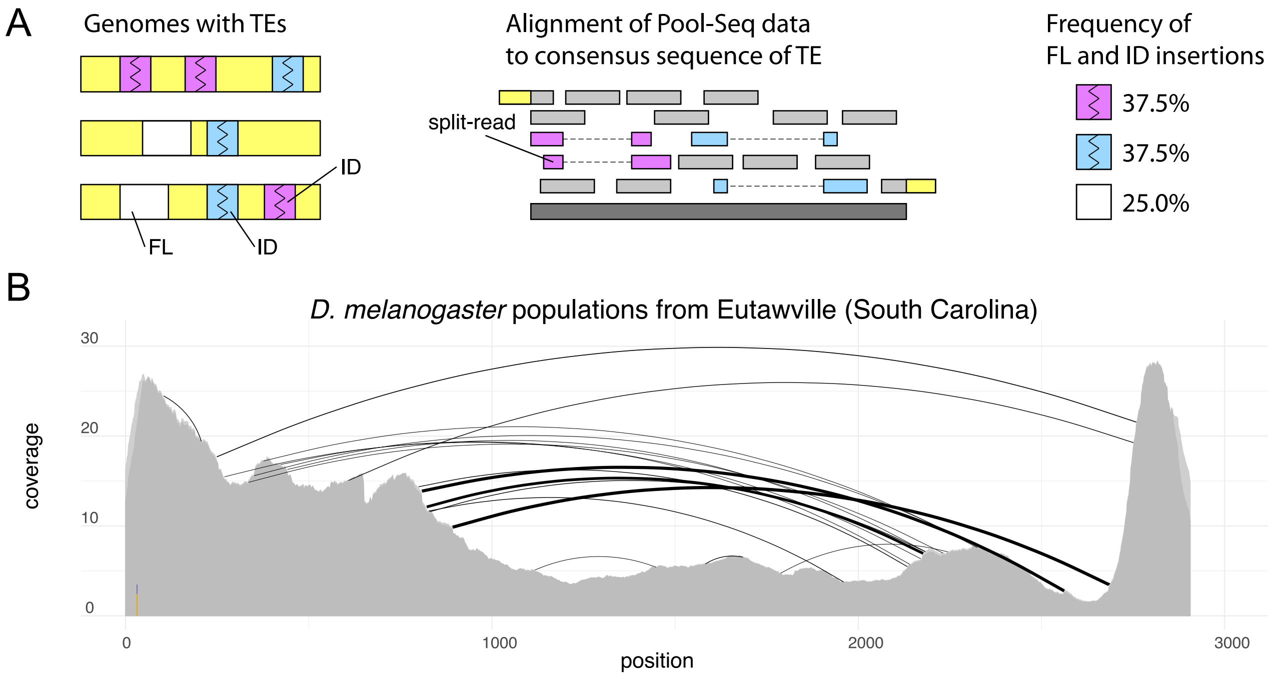
A
Genomes with TEs
ID
FL
ID
Alignment of Pool-Seq data
to consensus sequence of TE
split-read
Frequency of
FL and ID insertions
37.5%
37.5%
25.0%
B
D. melanogaster populations from Eutawville (South Carolina)
coverage
30
20
10
0
0
1000
2000
3000
position
