# Supplementary material for: Reconstructing the Invasion Route of the P-Element in Drosophila melanogaster Using Extant Population Samples
Source: Genome Biol Evol. 2020 Sep 10;12(11):2139–52. doi: 10.1093/gbe/evaa190 (PMC7750958; doi:10.1093/gbe/evaa190)
Supplement: evaa190_Supplementary_Data [file evaa190_supplementary_data.zip › HistoryOfPInvasion_forGBE.zip!/HistoryOfPInvasion/figures_vector/fig5-sim-deme_v2.pdf]

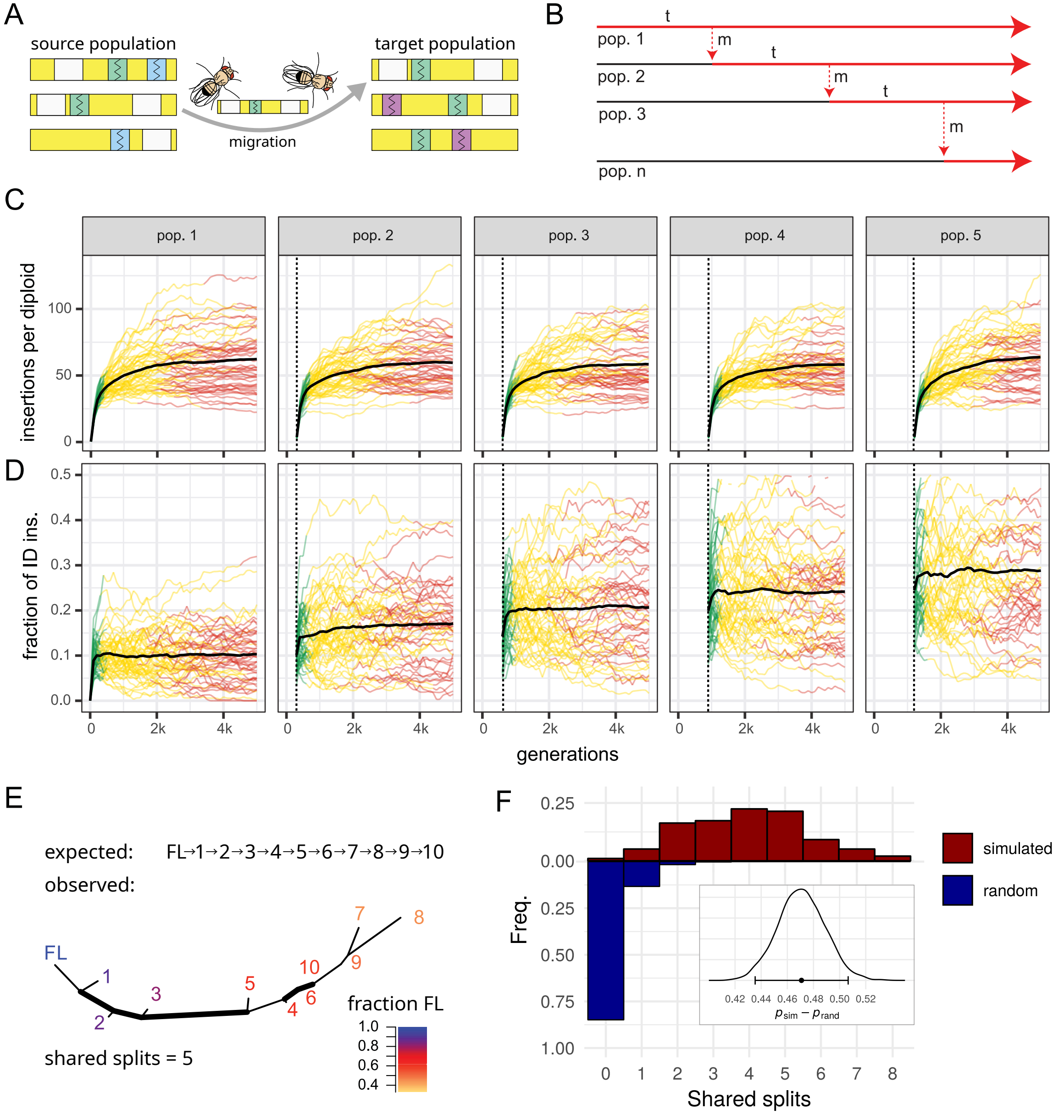

A
source population
migration
target population
B
t
pop. 1
m
t
pop. 2
m
t
pop. 3
m
pop. n
C
pop. 1
pop. 2
pop. 3
pop. 4
pop. 5
insertions per diploid
100
50
0
D
fraction of ID ins.
0.5
0.4
0.3
0.2
0.1
0.0
0
2k
4k
generations
E
expected: FL→1→2→3→4→5→6→7→8→9→10
observed:
FL
1
2
3
5
10
4
6
9
7
8
fraction FL
1.0
0.8
0.6
0.4
shared splits = 5
F
Freq.
0.25
0.00
0.25
0.50
0.75
1.00
simulated
random
0.42 0.44 0.46 0.48 0.50 0.52
$p_{sim} - p_{rand}$
Shared splits
0 1 2 3 4 5 6 7 8
